# A critical role for lymphatic endothelial heparan sulfate in lymph node metastasis

**DOI:** 10.1186/1476-4598-9-316

**Published:** 2010-12-20

**Authors:** Xin Yin, Jadwiga Truty, Roger Lawrence, Scott C Johns, R Sathish Srinivasan, Tracy M Handel, Mark M Fuster

**Affiliations:** 1 Department of Medicine, Division of Pulmonary and Critical Care, University of California San Diego, La Jolla, CA 92037 USA; 2 VA San Diego Healthcare System, San Diego, CA 92161 USA; 3 Department of Cellular and Molecular Medicine, Glycobiology Research and Training Center, University of California San Diego, La Jolla, CA 92093 USA; 4 Department of Genetics and Tumor Cell Biology, St. Jude Children's Research Hospital, Memphis, TN 38105 USA; 5 School of Pharmacy and Pharmaceutical Science, University of California San Diego, La Jolla, CA 92093, USA

## Abstract

**Background:**

Lymph node metastasis constitutes a key event in tumor progression. The molecular control of this process is poorly understood. Heparan sulfate is a linear polysaccharide consisting of unique sulfate-modified disaccharide repeats that allow the glycan to bind a variety of proteins, including chemokines. While some chemokines may drive lymphatic trafficking of tumor cells, the functional and genetic importance of heparan sulfate as a possible mediator of chemokine actions in lymphatic metastasis has not been reported.

**Results:**

We applied a loss-of-function genetic approach employing lymphatic endothelial conditional mutations in heparan sulfate biosynthesis to study the effects on tumor-lymphatic trafficking and lymph node metastasis. Lymphatic endothelial deficiency in *N-deacetylase/N-sulfotransferase-1 *(*Ndst1*), a key enzyme involved in sulfating nascent heparan sulfate chains, resulted in altered lymph node metastasis in tumor-bearing gene targeted mice. This occurred in mice harboring either a pan-endothelial *Ndst1 *mutation or an inducible lymphatic-endothelial specific mutation in *Ndst1*. In addition to a marked reduction in tumor metastases to the regional lymph nodes in mutant mice, specific immuno-localization of CCL21, a heparin-binding chemokine known to regulate leukocyte and possibly tumor-cell traffic, showed a marked reduction in its ability to associate with tumor cells in mutant lymph nodes. In vitro modified chemotaxis studies targeting heparan sulfate biosynthesis in lymphatic endothelial cells revealed that heparan sulfate secreted by lymphatic endothelium is required for CCL21-dependent directional migration of murine as well as human lung carcinoma cells toward the targeted lymphatic endothelium. Lymphatic heparan sulfate was also required for binding of CCL21 to its receptor CCR7 on tumor cells as well as the activation of migration signaling pathways in tumor cells exposed to lymphatic conditioned medium. Finally, lymphatic cell-surface heparan sulfate facilitated receptor-dependent binding and concentration of CCL21 on the lymphatic endothelium, thereby serving as a mechanism to generate lymphatic chemokine gradients.

**Conclusions:**

This work demonstrates the genetic importance of host lymphatic heparan sulfate in mediating chemokine dependent tumor-cell traffic in the lymphatic microenvironment. The impact on chemokine dependent lymphatic metastasis may guide novel therapeutic strategies.

## Background

Lymphatic metastasis in carcinoma is a major and early step during tumor progression, and its presence is a key determinant of cancer staging, treatment, and prognosis [1-3]. The process is characterized by pathophysiologic events that include local migration/invasion of tumor into lymphatic vessels, transport and survival within the lymphatic lumen, and colonization of regional lymph nodes [4,5]. In cancer, lymphatic remodeling and tumor invasion into the lymphatic conduit contribute directly to lymph node metastasis [6,7], and tumor-derived growth factors such as VEGF-C as well as chemokines produced by lymphatic vessels, such as CCL21/SLC (secondary lymphoid chemokine), have recently been implicated as key mediators of such events [8,9]. Recent studies have also shown that the chemokine receptor CCR7, which regulates lymphatic trafficking of leukocytes, including dendritic cells and T-cells, may contribute to lymph node metastasis of various cancers through interaction with its two known ligands, CCL21 and CCL19 [10-12]. Since CCL21 is produced by lymphatic endothelium, and known to drive lymphatic-directed immune cell traffic [13,14], it has been hypothesized that tumor cells may usurp this pathway in order to drive lymphatic invasion by over-expressing the CCR7 receptor [8,9,15]. The latter has been shown to correlate with nodal metastasis in human neoplasia [10,16,17]. More generally CCL21 may work in synergistic and redundant manners with other chemokines produced by lymphatic endothelium to facilitate lymphatic invasion by tumor cells that over-express chemokine cognate receptors [15,18]. Herein, we report the discovery of a central role played by unique glycans in regulating chemokine-mediated carcinoma cell traffic in vivo and in vitro, and we focus on CCL21 as a prototypical chemokine regulated by this novel process.

Recent studies have revealed a variety of co-receptor molecules that may regulate the coordinated actions of multiple lymphatic growth factors and/or chemokines during tumor lymphatic progression and metastasis. These include neuropilin-2 [19], integrins [20,21], and proteoglycans. The latter comprise a broad and ubiquitous class of glycoconjugates that play important roles in matrix and vascular remodeling, including the progression of such events in neoplasia (reviewed in [22,23]). By virtue of uniquely sulfated heparan sulfate (HS) glycan chains tethered to HS proteoglycan core proteins distributed over cell surfaces and the extracellular matrix (ECM), a variety of soluble proteins endowed with basic amino acid-rich domains, including growth factors and chemokines [24-26], may interact with negatively charged sulfate motifs on HS so as to form spatial gradients that may be biased by expression and distribution of the glycan. Heparan sulfate proteoglycans have been identified on lymphatic vessels [27]; and some chemokines, such as CCL21 and CXCL12, have been found to associate with both HS [28-30] as well as lymphatic endothelium during inflammation [18,31]. In neoplasia, HS may support chemokine gradients on the lymphatic vessel surface and ECM; however, direct in vivo evidence for this is lacking. Moreover, vascular chemokine signaling, which is influenced by rapid wash-out of chemokine as a result of blood- (or lymph) flow [32], may require matrix-associated HS to not only tether gradients of the relevant chemokines under flow, but also to critically support chemokine clustering and oligomerization required for optimal receptor binding and activation [26,32].

To examine the importance of the fine structure of HS in mediating tumor cell traffic in the lymphatic microenvironment, we genetically targeted lymphatic HS biosynthesis, and examined the effects on tumor-lymphatic chemo-attraction as well as lymph node trafficking in gene-targeted mice. Heparan sulfate is a linear polysaccharide that is covalently attached to either cell-surface bound or secreted proteoglycan core proteins. Structurally, HS is closely related to heparin, consisting of repeated disaccharide units of alternating uronic acid [D-glucuronic acid (GlcA) or L-iduronic acid (IdoA)] and D-glucosamine (GlcN) units [33]. As a result of unique modifications, including acetylation or sulfation on the amino group, O-sulfation at the 3- and 6-positions of GlcN, epimerization of GlcA to IdoA, and 2-O-sulation of IdoA, HS exhibits diverse structures with diverse functions [33,34]. The fine structure is precisely regulated by distinct chain-initiating enzymes (e.g., xylosyltransferase [XylT], which initiates sugar assembly on proteoglycan core proteins), polymerizing enzymes, and sulfate-modifying enzymes. The latter include the N-deacetylase/N-sulfotransferase (Ndst) family of isoenzymes, which initiates sulfation of nascent HS chains at discrete sites along the chain [22]. The sulfate modifications endow HS with the ability to bind and regulate the actions of growth factors as well as chemokines, such as interleukin 8 (IL-8) and stromal-derived factor-1α (CXCL12/SDF-1α), among others [35]. While a few HS-chemokine binding interactions have been characterized, including demonstration of altered heparin binding as a result of targeting specific basic amino acid - rich protein domains of certain chemokines [26], the biological importance of targeting the presence or biosynthesis of the glycan in chemokine-dependent tumor cell trafficking, including lymph node metastasis, has never been reported.

In this study, we report that genetic alteration of lymphatic endothelial HS disrupts chemokine-dependent migration of tumor cells toward the targeted endothelium; and deletion of Ndst1 in the lymphatic endothelium in vivo is sufficient to alter lymph node colonization by tumor. In cell-based studies, we investigate mechanisms that may underlie these findings, and show that CCL21-dependent tumor cell migration toward lymphatic endothelium critically depends upon lymphatic HS. We demonstrate that targeted disruption of HS produced into the conditioned medium of lymphatic endothelia is sufficient to abrogate CCL21-CCR7 binding as well as the activation of migration signaling pathways in tumor cells exposed to the same medium. In addition, we show that lymphatic cell-surface HS may serve as a source for the establishment of CCL21 gradients that drive tumor cell migration. These findings suggest key roles for lymphatic HS during the trafficking of tumor cells and colonization of lymph nodes in carcinoma.

## Results

### Lymphatic endothelial HS is required for directional invasion of tumor cells across matrix

**Figure 1:**
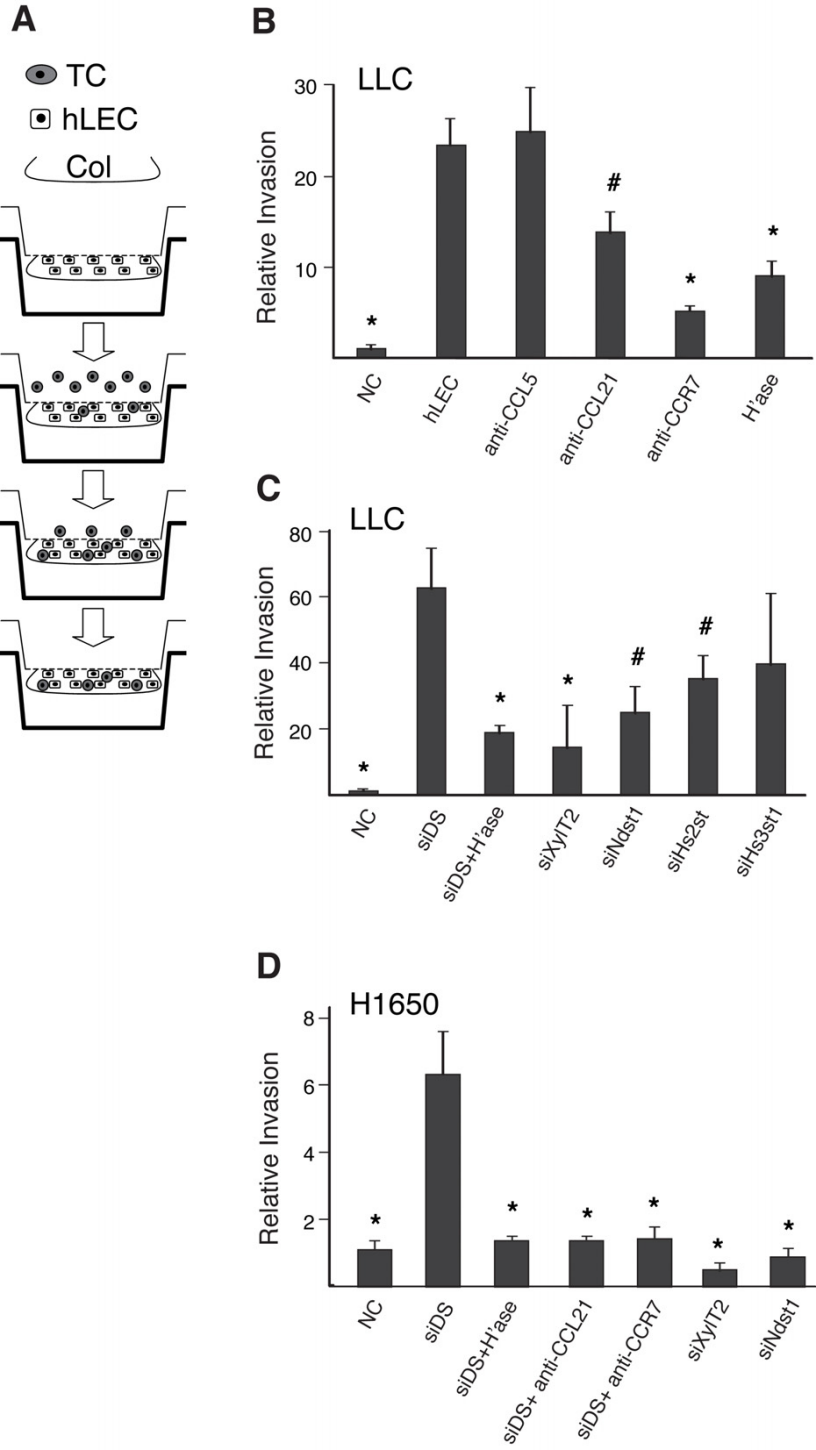
**Invasion of tumor cells toward matrix-embedded lymphatic endothelial cells depends on lymphatic heparan sulfate**. **A**. Schematic representation of a modified transwell collagen invasion assay. TC, tumor cells; hLEC, primay human lung lymphatic endothelial cells; Col, type I collagen gel. **B **and **C**. Invasion of Lewis lung carcinoma cells (LLC) toward collagen gel containing either no cells ("NC") or hLEC treated as indicated was quantified and normalized to NC. αCCL21, αCCR7 or αCCL5, blocking antibodies to CCL21, CCR7 or CCL5, respectively; H'ase, hLEC pre-treated with heparinase; siDS, hLEC transfected with control (scrambled duplex) RNA; siNdst1, siXylT2, siHs2st or siHs3st1, hLEC transfected with siRNA targeting corresponding HS biosynthetic enzymes. **D**. Invasion of human lung adenocarcinoma cells (H1650) toward collagen gel containing either no cells ("NC") or hLEC treated as indicated was quantified and normalized to NC. *P < 0.01, ^#^*P *< 0.05, as compared to hLEC in **B **and siDS in **C **and **D**.

To test whether HS produced by lymphatic endothelial cells in collagen matrix plays a role in attracting the invasion of tumor cells in vitro, we designed a matrix-based invasion assay: Primary human lymphatic endothelial cells (hLEC) were embedded in type-I collagen on the underside of transwell filters, and either murine Lewis lung carcinoma cells (LLCs) stably expressing GFP or human lung carcinoma (H1650) cells labeled with fluorophore (calcein) were loaded on top of the insert. Tumor cells were allowed to invade into the collagen gel, and gel-incorporated tumor cells were quantified in the setting of unique conditions targeting HS produced by the hLEC (Figure 1). Two targeting approaches were applied to target lymphatic endothelial HS in the system: (1) Enzymatic pre-treatment of the hLEC with heparinase (which destroys HS chains [36]), and (2) altering HS production via pre-treatment of the hLEC with siRNA targeting HS biosynthetic enzymes. Transient transfection of the hLEC with siRNA resulted in approximately 80% knockdown of targeted mRNAs, and ~70% reduction at the protein level, as compared to control transfection of hLEC with scrambled-duplex RNA (siDS) (Additional File 1 - Figure S1).

The presence of control hLEC in the collagen was able to drive invasion by LLCs (Figure 1B, compare invasion toward "hLEC" control cells to that toward "NC" (no hLEC) negative control)). The presence of blocking antibodies to either CCL21 or CCR7 significantly reduced invasion; and the presence of intact HS on the hLEC was necessary for LLC invasion, as evidenced by marked inhibition upon treating the hLEC with heparinase, which destroys HS. Targeting the HS sulfating enzymes Ndst1 or Hs2st (responsible for glucuronyl N-sulfation or iduronyl 2-O-sulfation of HS, respectively), also significantly reduced LLC invasion (Figure 1C). In contrast, antibody blockade of CCL5 (Figure 1B) or treatment with siRNA targeting a distinct HS sulfating enzyme (i.e., Hs3st1 (Figure 1C) responsible for glucoronyl 3-O-sulfation of HS) did not significantly inhibit invasion, suggesting distinct and specific requirements with respect to chemokines as well as the fine structure of lymphatic endothelial HS in the system. Targeting lymphatic endothelial HS during invasion by human lung adenocarcinoma (H1650) cells also abrogated the ability of the tumor cells to invade (Figure 1D).

### Genetic targeting of pan-endothelial HS biosynthesis alters the spread of Lewis lung carcinoma to regional lymph nodes

**Figure 2:**
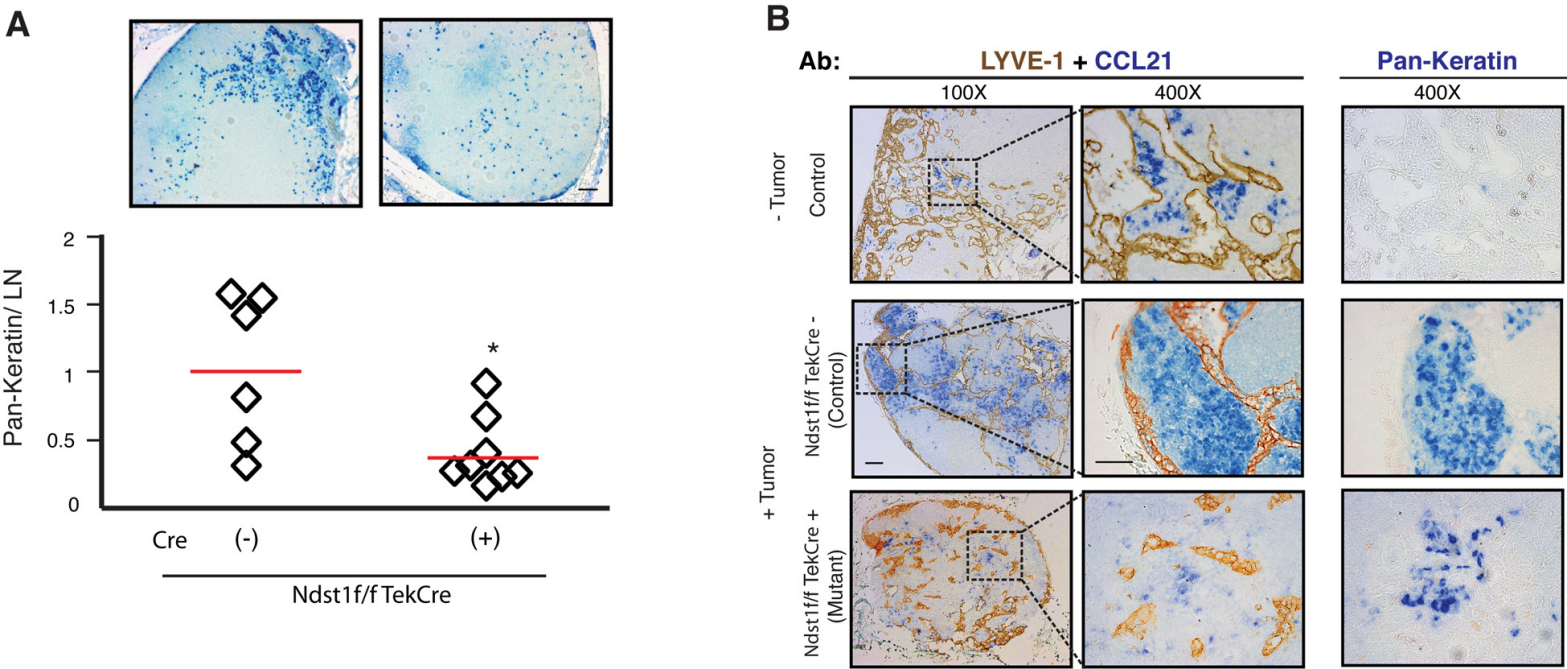
**Genetic targeting of pan-endothelial heparan sulfate biosynthesis impairs metastasis of carcinoma to regional lymph nodes**. LLC tumor cells were injected subcutaneously into the left caudal/medial inguinal region of *Ndst1*^f/f^*TekCre+ *mutant mice, which bear a pan-endothelial mutation in the major HS sulfating enzyme *Ndst1*, and their wildtype *Cre- *littermates as controls. After 14 days, the left subiliac lymph node (draining the primary tumor) from each mouse was isolated. **A**. Metastaic tumor cells in the lymph nodes (LN) were detected using anti-pan-keratin antibody (blue stain) on LN tissue sections imaged under 100× magnification, and quantified (NIH Image-J) to determine net pixel intensity for each LN. Values were normalized to the mean pixel value/LN among control (*Ndst1*^f/f^*TekCre-*) littermates. Graph is shown below; *P = 0.016, for difference in mean values (red bars) for mutant (N = 8) vs control (N = 6) mice. **B**. Representative LN tissue sections from tumor-bearing mutant (bottom row of photomicrographs) vs wildtype (*Cre*^-^) controls (middle row) were co-stained with CCL21 (blue) and LYVE1 (brown) (left two panels) or pan-keratin (blue, right panel). The top row of photomicrographs shows a representative LN section from a non-challenged (tumor free) control mouse. Scale bars, 100 μm for 100× and 50 μm for 400× magnification photomicrographs.

The initial in vitro findings prompted us to target lymphatic HS biosynthesis in vivo, and examine how that might affect colonization of regional lymph nodes by tumor cells. In a preliminary model, Lewis lung carcinoma (LLC) cells (syngeneic on the C57Bl/6 background) were subcutaneously injected into the left caudal/medial inguinal fold of *Ndst1*^f/f^*TekCre*^+ ^mutant mice (N = 8) and their *Cre*^- ^wildtype littermates (N = 6) to induce tumors. Mutants have a pan-endothelial deficiency in the major HS sulfating enzyme Ndst1 [37,38]. Knockdown was confirmed in primary lymphatic endothelial cells (LEC) purified from mutant versus wildtype littermates, wherein deletion efficiency of the loxP-flanked allele of *Ndst1 *in cultured primary LEC from *Cre*^+ ^mutants was found to be ~75% by quantitative genomic PCR, consistent with the known lymphatic endothelial expression of the *Tek *promoter in vivo [39,40]. When primary LLC tumor size reached ~0.5 cm in diameter (where primary tumor size was comparable among mutants and wildtype littermates), the regional subiliac lymph node from each mouse was isolated, and nodal tumor mestastasis was examined using pan-keratin immunohistochemical (IHC) staining (Figure 2).

Colonization of lymph nodes by tumor in the mutants was markedly reduced compared to that in *Cre*^- ^littermates (comparing lymph node pan-keratin immunoreactivity; Figure 2A), suggesting that appropriate sulfation of lymphatic endothelial HS was essential for lymphatic metastasis. With respect to CCL21: Lymph nodes from wildtype mice that were not challenged with tumor were characterized by minimal CCL21 immunoreactivity distributed in the matrix surrounding LYVE-1-positive lymphatic vessels (Figure 2B, top panels). In tumor-challenged wildtype littermates, large areas of CCL21 positive cells were detected throughout the lymph node cortices, and pan-keratin positive patches of metastatic tumor cells in the lymph nodes were co-localized with CCL21 immunoreactivity (Figure 2B, middle row). However, in *Cre*+ mutants, pan-keratin positive tumor patches were markedly reduced (Figure 2A, graph and photomicrographs), and detectable tumor deposits were characterized by minimal associated CCL21 immunoreactivity (Figure 2B, bottom row). In the course of LYVE-1 staining, we noted that the LLC primary tumor was characterized by an absence of intra-tumoral LYVE1-positive lymphatic vessels in wildtype mice; and at the given primary tumor size (0.5 cm diameter), the density of sparse lymphatic vessels along the periphery of tumors from mutant versus wildtype littermates did not significantly differ (data not shown).

### Lymphatic endothelial conditioned medium is sufficient to drive chemokine-dependent tumor cell migration, and altering lymphatic HS in the medium disrupts migration

**Figure 3:**
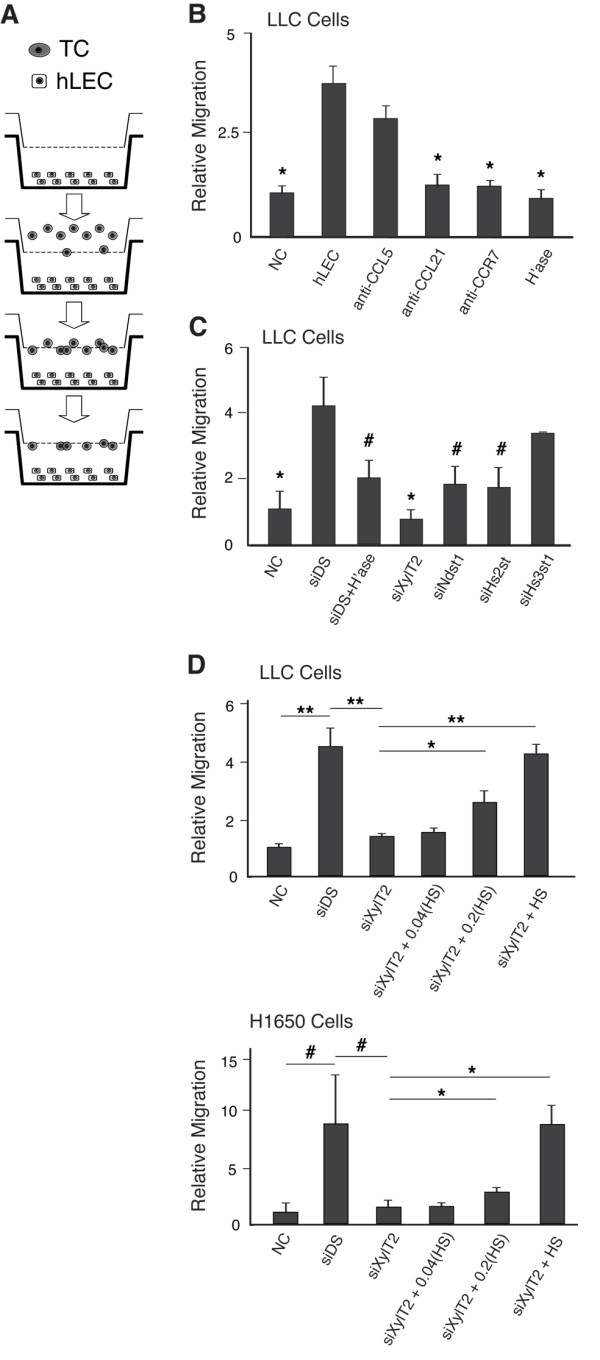
**Migration of Lewis lung carcinoma cells toward lymphatic endothelial cells depends on lymphatic heparan sulfate**. **A**. Schematic representation of a modified transwell chemotaxis assay, wherein liquid medium separates migrating LLC tumor cells that initiate in the upper well and migrate toward lower wells that contain either no cells or hLEC monolayers treated under various conditions. **B **and **C**. Transwell migration of tumor cells into lower wells plated with either no cells as a negative control ("NC") or hLEC monolayers treated as indicated was quantified, and plotted for each condition as the mean -fold response over the value for NC (and normalized to the NC value). Conditions in **B **refer to the addition of specific blocking antibodies or pre-treatment of the hLEC with heparinase (H'ase); and conditions in **C **refer to treatment of the hLEC with siRNA targeting the indicated HS biosynthetic enzymes. *P < 0.01, ^#^*P *< 0.05, as compared to hLEC group in **B **and siDS group in **C**. **D**. Graphs quantifying transwell migration of either LLC (upper graph) or H1650 (lower graph) lung carcinoma cells into lower wells plated with either no cells (NC), control hLEC (siDS), or siXylT2 targeted hLEC. The condition "siXylT2+HS" (far right) refers to addition of heparan sulfate purified from cultured control hLEC into the lower-well medium during tumor cell migration toward siXylT2 targeted hLEC. Fractions (0.04 and 0.2) of the total HS needed for rescue were used in separate wells to test dose-response. ^#^*P *< 0.05, *P < 0.01, **P < 0.001 for histogram comparisons indicated by horizontal bars.

We next examined whether HS secreted by lymphatic endothelial cells might facilitate chemokine-dependent tumor cell migration toward lymphatic endothelium. To study this, LLC tumor cells on the upper side of transwell filters were separated from HS-targeted hLEC monolayers in the lower wells by liquid medium (Figure 3A), and LLC migration into the bottom wells was quantified. The presence of hLEC in the bottom well was sufficient to drive LLC migration (Figure 3B), and blocking either CCL21/CCR7 (Figure 3B) or interfering with the biosynthesis of hLEC HS (Figure 3C; using siRNA targeting XylT2, Ndst1, or Hs2st) significantly reduced LLC migration. In contrast, CCL5 blocking antibody (Figure 3B) or treatment of hLEC with Hs3st1 siRNA (Figure 3C) was not sufficient to alter migration, suggesting that the presence- as well as specific sulfation properties of HS produced in the hLEC conditioned medium is required for chemokine-dependent migration of LLC cells toward the hLEC. Moreover, to further demonstrate the specific importance of *trans*-acting secreted lymphatic HS in this process, addition of purified lymphatic endothelial HS back into the medium rescued the alteration in tumor migration resulting from silencing hLEC XylT2 (which initiates HS chain biosynthesis) in a dose-dependent manner (Figure 3D). This was carried out for invasion by both LLC cells and H1650 lung carcinoma cells.

### Heparan sulfate secreted by lymphatic endothelium activates CCL21-dependent tumor cell migration signaling pathways *in trans*

**Figure 4:**
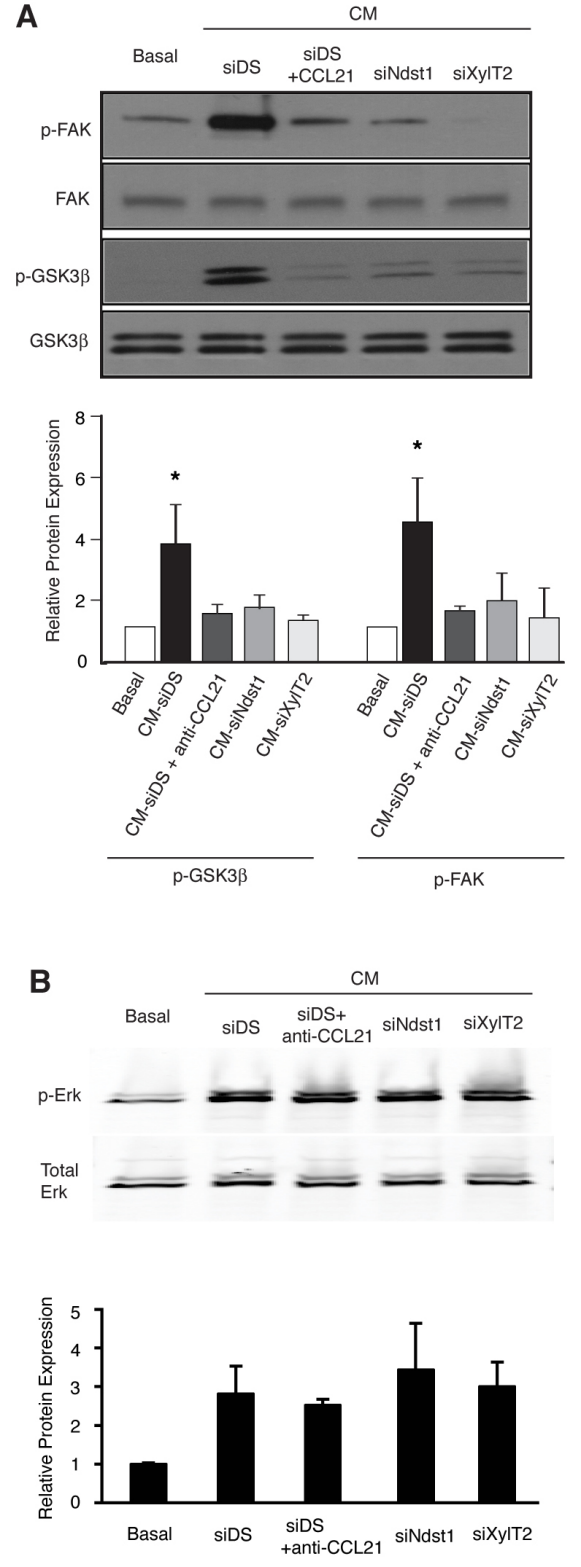
**Lymphatic-secreted HS activates tumor-cell migration signaling pathways *in trans***. **A**. Serum-free basal endothelial growth medium or conditioned medium (CM) harvested from hLEC treated as indicated was applied to cultured LLC for 10 min. The LLC were lysed and the indicated target proteins were detected by Western immunoblotting. A representative gel image (top) as well as normalized densitometric quantification (below) from three independent experiments is shown. Relative protein expression was calculated as the densitometric ratio of phosphorylated protein to that of corresponding total protein, and normalized to the basal value. *P < 0.05, as compared to LLC treated with CM from control hLEC (CM-siDS). **B**. In separate experiments, the effects of blocking CCL21 (siDS + αCCL21) or treatments that alter HS biosynthesis (siNdst1 or siXylT2) on CM-mediated Erk1/2 phosphorylation were examined.

We examined whether genetic alteration of lymphatic-secreted HS might (*in-trans*) play a key role in mediating activation of migration signaling in the tumor cells. Conditioned medium (CM) harvested from HS-targeted hLEC was applied to cultured LLC cells, and the cells were examined for the activation of two migration-related signaling intermediates: focal adhesion kinase (FAK) and glycogen synthase kinase 3β (GSK3β) (Figure 4A), both of which are activated in migrating tumor cells through phosphorylation. Compared to LLC exposure to basal growth medium, exposure to CM harvested from control (siDS) hLEC led to robust FAK and GSK3β phosphorylation. Exposure to CM from control hLEC in the presence of CCL21 blocking antibody, or to CM collected from siNdst1- or siXylT2-targeted hLEC, abrogated phosphorylation of the kinases (see also quantitative data in Figure 4A). Interestingly, altering lymphatic HS in the CM did not appear to alter the phosphorylation of mitogen activated protein kinase Erk1/2 in tumor cells in response to CM exposure (Figure 4B), indicating that targeting HS in the lymphatic CM may specifically affect migration signaling pathway activation in CM-exposed tumor cells.

### Lymphatic endothelial HS serves as a co-receptor for chemokine-receptor binding on the surface of tumor cells, and altering HS sulfation alters chemokine-receptor association

**Figure 5:**
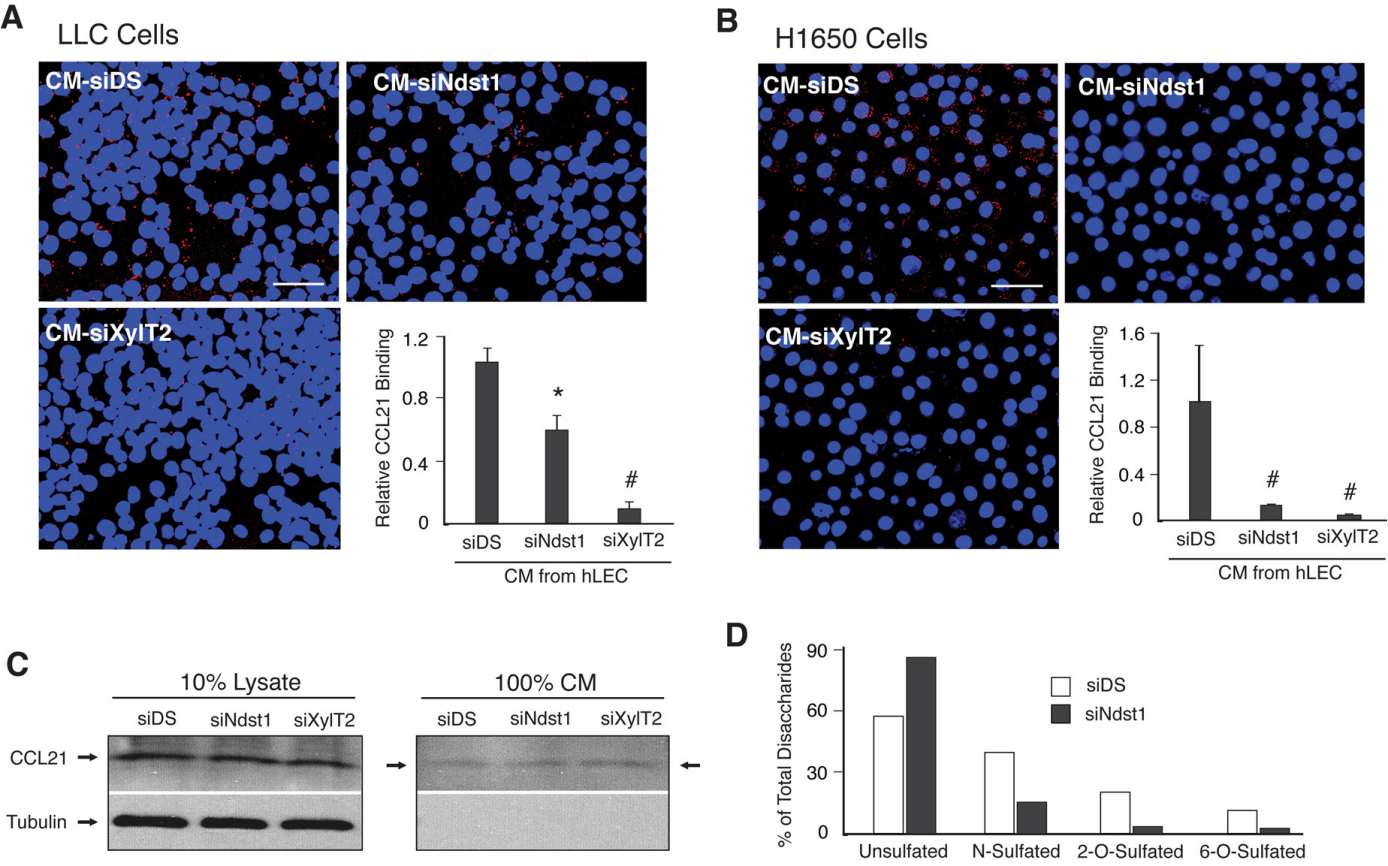
**Heparan sulfate in the lymphatic conditioned medium is required for CCL21 binding to tumor cells**. Conditioned medium (CM) from hLEC transfected with either control (scrambled duplex) RNA (siDS) or siRNA targeting the indicated HS biosynthetic enzymes was harvested and applied to LLCs (**A**) or H1650 (**B**) cytospin samples, respectively. Binding of CCL21 in the CM to CCR7 on LLC was detected by proximity ligation assay (PLA). Representative merged images showing PLA signal (red) and nuclear DAPI stain (blue) were taken by fluorescence microscopy (400 ×)(**A **and **B **photomicrographs; Scale bar, 50 μm.) PLA signal from each field was quantified and indexed to total nuclear area within the same field (**A **and **B **graphs). At least 5 random fields from each group were included for analysis. Mean data was normalized to control signal (CM-siDS). *P < 0.05, ^#^*P *< 0.01, as compared to CM-siDS group. **C**. For each hLEC siRNA treatment condition, CCL21 in both the cell lysate (left) and CM were detected by Western immunoblot analysis. Tubulin was probed as a loading control. **D**. HS was purified from CM of siDS- or siNdst1-transfected hLEC and the sulfation status examined by disaccharide analysis using liquid chromatography/mass spectrometry.

We examined whether HS produced into CM of hLEC is required for binding of CCL21 to its receptor on lung carcinoma cells exposed to the medium. Specifically, CCL21-CCR7 association on carcinoma cells in the presence of CM harvested from control versus HS-targeted hLEC was examined. The analysis was carried out for both LLC tumor cells (Figure 5A) as well as human lung carcinoma cells (Figure 5B) using a proximity ligation assay (PLA) wherein the proximity of antibodies applied to primary ligand (CCL21) and receptor (CCR7) protein targets was measured through the use of novel bifunctional secondary antibodies. The assay generates a fluorescent signal only when the antibody-labeled ligand and receptor protein targets are in close proximity [41]. It should be noted that bound CCL21 detected in the assay results from presence of endogenous CCL21 in the CM (i.e., there was no addition of exogenous CCL21 in the assay). To test the CM from HS-targeted hLEC in the assay, HS biosynthesis in the hLEC was targeted through either siNdst1 (targeting HS sulfation) or siXylT2 (targeting HS chain initiation), and the hLEC conditioned media were collected. The engagement of numerous cell-surface CCL21:CCR7 complexes was noted upon exposure of LLCs to CM from control (scrambled-duplex RNA -treated) hLEC (Figure 5A; CM-siDS). On the other hand, reduction in CCL21:CCR7 complexes was noted upon exposure of LLCs to CM from siNdst1 targeted hLEC, and further reduction occurred upon exposing the cells to siXylT2-targeted CM (Figure 5A, graph). The exposure of human H1650 lung carcinoma cells to the CM from siNdst1- or siXylT2-targeted hLEC also resulted in abrogation of CCL21:CCR7 association on the tumor cell surface under similar conditions (Figure 5B).

In separate tests, to examine whether targeting lymphatic HS biosynthesis might alter the production of chemokine by the hLEC, the levels of CCL21 produced by control (siDS) vs HS-targeted hLEC were examined. Targeting HS biosynthesis in the hLEC using siNdst1 and siXylT2 did not reduce the expression of CCL21 by the hLEC (Additional File 1 - Figure S1); and the protein levels of CCL21 measured in the CM as well as cell lysates of siNdst1 or siXylT2 targeted hLEC were not reduced relative to that of control (siDS) (Figure 5C). These data suggested that reduced receptor-dependent binding of CCL21 to tumor cells was not simply caused by reduced production or availability of CCL21. Thus, HS secreted by the hLEC might serve an essential function in mediating specific presentation of soluble lymphatic chemokine *in trans *to cognate receptor on the tumor cells, including critical clustering/oligomerization of chemokines that may be necessary for receptor binding [26,32]. To confirm whether siRNA targeting of Ndst1 in the hLEC was sufficient to reduce sulfation of HS glycan chains produced in the CM, we analyzed the sulfate composition of HS purified from the conditioned media of siDS vs siNdst1-targeted hLEC by mass spectrometry. HS disaccharides purified from the CM of siNdst1-targeted hLEC showed marked reductions in N-, 2-O-, and 6-O-sulfation as compared to that of control (siDS) hLEC (Figure 5D; see also Additional File 2 - Figure S2 for detailed sulfation analysis by disaccharide species). Accordingly, HS purified from siNdst1-targeted hLEC was characterized by an increase in the percentage of unsulfated disaccharides as compared to that of control hLEC.

### Lymphatic endothelial cell-surface HS may establish chemokine gradients by tethering CCL21 to the lymphatic endothelial surface

**Figure 6:**
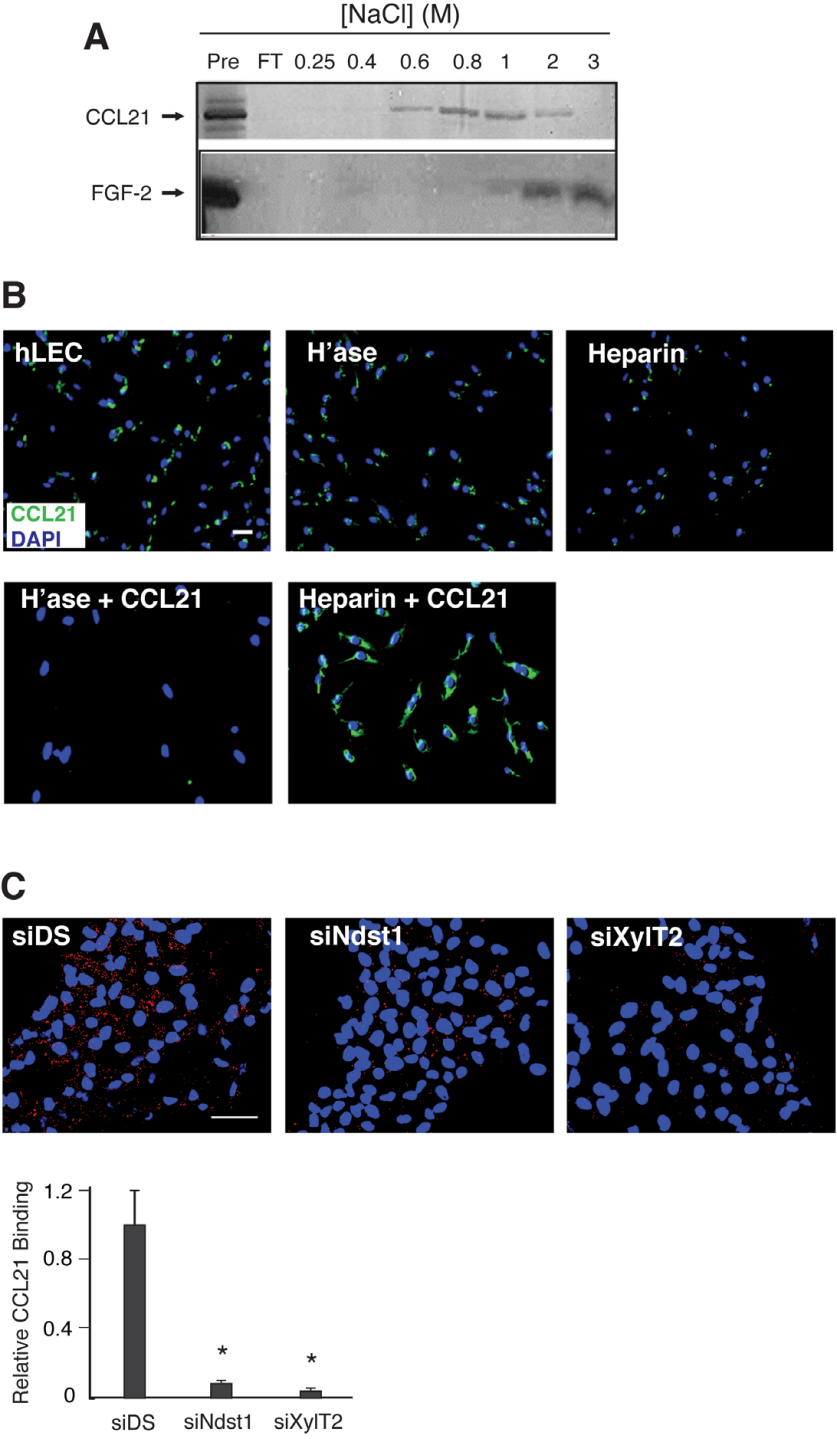
**Lymphatic endothelial heparan sulfate is required for receptor-dependent display of CCL21 on the lymphatic surface**. **A**. Binding of human CCL21 (hCCL21) to heparin was examined using heparin affinity chromatography followed by silver staining of salt-eluted fractions on SDS-PAGE (upper panel). "Pre," recombinant human CCL21 sample directly loaded onto the silver-stained gel; "FT," flow-through from the column. Basic fibroblast growth factor (FGF-2) was run as a positive control (lower panel). **B**. hLEC were treated as indicated and the binding of CCL21 to hLEC was examined by immunofluorescence (IF) using anti-CCL21 antibody. hLEC, untreated cells; H'ase, Heparin: hLEC pre-treated with heparinase or heparin, respectively; H'ase+CCL21, Heparin+CCL21: hLEC pre-treated with heparinase or heparin, respectively, followed by addition of exogenous human recombinant CCL21 (+CCL21). Representative merged images showing CCL21 signal (green) and DAPI nuclear stain (blue) were taken under 100× magnification. (Scale bar, 100 μm.) **C**. hLEC were transfected with either control RNA (siDS) or siRNA targeting HS biosynthetic enzymes *Ndst1 *or *XylT2*. Binding CCL21 to CCR7 on hLEC was detected by proximity ligation assay (PLA). Representative merged images showing PLA signal (red) and nuclear DAPI stain (blue), imaged using fluorescence microscopy (400×; Scale bar, 50 μm). PLA signal from each field was quantified and indexed to total nuclear area within the same field, and mean values for each condition were graphed (bottom). At least 5 random fields from each group were included for analysis. Mean data was normalized to control signal (siDS). *P < 0.01, as compared to control.

Heparan sulfate produced on the surface of lymphatic endothelial cells may serve as a source for tethering gradients of lymphatic chemokine(s). To test this hypothesis, we examined the presentation of CCL21 on the surface of cultured hLEC. We initially carried out heparin affinity chromatography to assess the degree to which CCL21 binds to heparin-sepharose columns (Figure 6A). A moderate-to-high degree of salt (0.8 - 1.0 M NaCl) was required to elute recombinant human CCL21 from the column. Recombinant human FGF-2, run as a known strong heparin-binding growth factor, required 2 - 3 M NaCl for elution. In separate studies, we also used a plate-based method to characterize the binding interaction of CCL21 with plate-bound heparin (Additional File 3 - Figure S3), which showed a Kd (8.3 nM) similar to that previously reported for CCL21 interacting with immobilized heparin [42]. In cell-based studies, a basal level of endogenous CCL21 present on the hLEC surface, as detected by immunofluorescence, could be essentially cleared by treatment with heparinase (which destroys cell-surface HS), or by washing with heparin (Figure 6B, upper panels). Moreover, exogenous CCL21 could be "re-loaded" on the cell surface following heparin wash, while pre-treatment with heparinase rendered the cell surface incapable of binding exogenous CCL21 (Figure 6B, lower panels, "+CCL21"), suggesting that HS plays a key role in maintaining CCL21 (including endogenous levels) on the hLEC surface. In separate studies, we also found that hLEC express CCR7 (Additional File 4 - Figure S4). To specifically examine whether hLEC cell-surface HS might be required for CCL21-CCR7 association on hLEC, we performed PLA using CCL21 and CCR7 antibodies, and found that binding of endogenous CCL21 produced by the hLEC to CCR7 on the hLEC cell surface was sensitive to altering HS biosynthesis in the hLEC through either siNdst1 or siXylT2 targeting (Figure 6C). It is noteworthy that addition of exogenous recombinant CCL21 to control (siDS) hLEC resulted in a higher level of receptor-bound CCL21 than that of basal/endogenous CCL21; however, the difference fell short of statistical significance (*P *= 0.09), possibly because of a large number of CCR7 receptor sites already occupied by endogenous CCL21. Nevertheless, ablation of HS chain biosynthesis (i.e. targeting using siXylT2) also prevented the binding of exogenous CCL21 to the cells (data not shown).

### Genetic knock-down of lymphatic endothelial HS sulfation through a lymphatic-specific mutation in *Ndst1 *impairs the spread of Lewis lung carcinoma to regional lymph nodes

**Figure 7:**
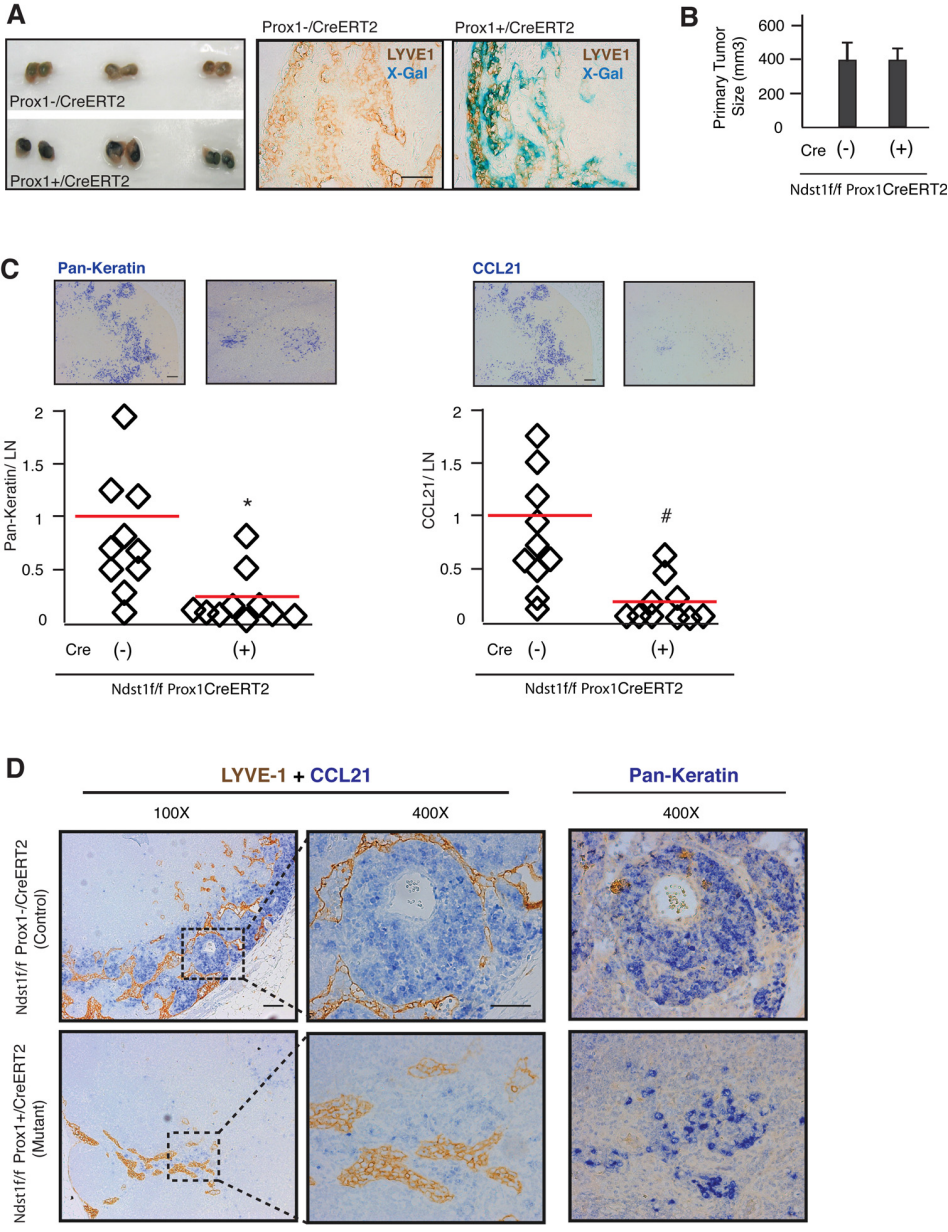
**Genetic alteration of lymphatic endothelial heparan sulfate biosynthesis impairs tumor metastasis to regional lymph nodes**. **A**. *Cre *reporter testing in the inducible *Prox1*^*+/CreERT2 *^model: Bilateral subiliac lymph nodes (LN) were isolated from *Prox1*^*+/CreERT2*^*Rosa26R *reporter mice ("*Prox1+/CreERT2*", lower panel) or their *Prox1*^*-/CreERT2*^*Rosa26R *littermates ("*Prox1-/CreERT2"*, upper panel) after intraperitoneal injection of Tamoxifen for 5 consecutive days; and stained with X-Gal (showing as deep blue stain in *Cre *positive lymph nodes). Right panels: subiliac LNs from **A **were sectioned and stained for LYVE-1 (brown) and X-Gal (blue); and imaged under 400 × magnification (Scale bar, 50 μm). For tumor establishment, LLC tumor cells were injected subcutaneously into the left caudal-medial inguinal region of *Ndst1*^f/f^*Prox1*^*+/CreERT2 *^mice (N = 10) and their *Ndst1*^f/f^*Prox1*^*-/CreERT2 *^wildtype littermates (N = 10) following a 5-day tamoxifen dosing schedule. Following 14 days of primary tumor growth, the left subiliac regional LN from each mouse was isolated. **B**. Comparison of primary tumor volumes from *Cre *postive vs *Cre *negative mice at time of experiment termination and lymph node harvesting. **C**. Representative photomicrographs of pan-keratin stained LN histologic sections are shown above plotted data on the left, which shows quantified pan-keratin signal per LN for each mouse. Graph to the right shows plotted data for degree of CCL21 immunoreactive signal per LN (and representative photomicrographs of CCL21 staining from Cre positive vs Cre negative animals above the graph). LN staining signal from both groups was imaged under 100× magnification, quantified as pixel values per LN using NIH Image J software, and normalized to the average pixel value/LN in *Ndst1*^f/f^*Prox1*^*-/CreERT2 *^control group. *P < 0.05, ^#^*P *< 0.01, for comparison of mean mutant vs control group values. **D**. LNs from both groups were co-stained with CCL21 (blue) and LYVE1 (brown) (left two panels) or pan-keratin alone as a marker for metastatic tumor cells (blue signal, right panel). Scale bars, 100 μm for 100× and 50 μm for 400× magnification.

Following in vitro results as well as the finding that a pan-endothelial mutation in *Ndst1 *was associated with reduced tumor colonization of regional lymph nodes in the LLC tumor model (Figure 2), we sought to confirm whether that result might have stemmed from specifically altering HS in the lymphatic endothelium. Lymphatic vasculature may carry tumor cells directly between primary tumor and the regional lymph node; however, in a pan-endothelial HS mutation model (i.e., tissue targeting using *TekCre*, as in Figure 2), the additional presence of the mutation in blood-vascular endothelium has the potential to alter HS biosynthesis in lymph node high endothelial venules (i.e., potential to also affect blood-borne lymph node metastasis). To address this and maximize genetic specificity in vivo, tumors were established by injecting LLC tumor cells into the left caudal/medial inguinal fold of *Ndst1*^f/f^*Prox1*^*+/CreERT2 *^mutant mice (N = 10) and *Ndst1*^f/f^*Prox1*^*-/CreERT2 *^wildtype littermates (N = 10), wherein the *Ndst1 *mutation could be inducibly driven under control of the *Prox1 *promoter, which is expressed exclusively in *lymphatic *endothelium [43]. In preliminary reporter studies, induction of *Cre *recombinase in the lymphatic endothelium of *Prox1*^*+/CreERT2 *^mutants following a schedule of tamoxifen (administered to both *Cre *positive and *Cre *negative control animals) was confirmed in lymphatic vessels, including those within lymph nodes, using a *Cre *reporter line (*Rosa26R*) bred onto the *Prox1*^*+/CreERT2 *^vs *Prox*^*-/CreERT2 *^backgrounds, and staining of tissues with X-gal (Figure 7A).

Following tumor induction in the compound (*Ndst1*^f/f^*Prox1*^*+/CreERT2*^) mutants and their wildtype *Cre*^- ^littermates, when the primary LLC tumor size reached ~0.5 cm in diameter (confirmed to be equivalent in both *Cre *positive mutants and *Cre *negative wildtype littermates; Figure 7B), we isolated regional subiliac lymph nodes, and analyzed nodal tumor metastases through IHC analysis of lymph node sections for pan-keratin. Mutant lymph nodes were characterized by a striking reduction in tumor deposits, as compared to that in *Cre *negative control littermates (Figure 7C, left graph), indicating that appropriately sulfated lymphatic endothelial HS is critically required for lymph node metastasis. It should be noted that this LLC tumor model was characterized by absence of intratumoral lymphangiogenesis in the primary tumors of wildtype mice; however, scant LYVE-1 positive lymphatic vessels were found surrounding the tumors at the time of lymph node harvest. Regional lymph nodes were characterized by lymphatic proliferation in tumor-bearing mice (with a mean 2.85 - fold increase in the total lymph node lymphatic conduit, as measured by LYVE-1 immunoreactivity, in tumor-associated lymph nodes as compared to non-tumor associated nodes; *P *= 0.004). Among tumor-bearing mice, lymphatic vessel density in the lymph nodes from *Ndst1 *mutants was lower than that of their wildtype littermates (Additional File 5 - Figure S5), although the difference fell short of meeting statistical significance. With respect to metastases in regional lymph nodes draining the tumors, however, there were striking differences in mutant versus wiltype lymph nodes. The regional lymph nodes from *Ndst1*^f/f^*Prox*^*-/CreERT2 *^wildtype littermates showed large areas of CCL21 positive cells that were found to tightly localize to areas of pan-keratin positive tumor metastases (Figure 7C, compare pan-keratin and CCL21 photomicrographs from a representative *Cre *negative animal, shown above graphs). Some CCL21 staining showed spotty co-localization with LYVE-1 along the walls of lymphatic vessels in the lymph nodes (Additional File 6 - Figure S6), although most immunolocalization of the chemokine ligand was associated with metastatic tumor cells. On the other hand, in lymph nodes of *Ndst1*^f/f^*Prox1*^*+/CreERT2 *^mutants, regional lymph nodes were characterized by not only minimal pan-keratin positive tumor deposits (Figure 7C, left graph); but in contrast to wildtype littermates, the chemokine showed markedly reduced presence/localization of CCL21 with pan-keratin positive tumor areas (Figure 7C, compare representative photomicrographs from *Cre *positive animal above graphs; and Figure 7D, showing another example at 100× and 400×). This suggested that the specific loss of Ndst1 in the lymphatic endothelium reduced not only lymph node metastasis, but also the ability of CCL21 to associate with tumor cells in the lymph node sections.

## Discussion

In this study, we demonstrate that altering HS biosynthesis in the lymphatic endothelium *in vivo *results in altered regional lymph node colonization by tumor cells in experimental mouse carcinomas. In cell-based studies, targeted disruption of lymphatic endothelial HS biosynthesis altered the ability of the lymphatic endothelium to attract tumor cells in a chemokine-dependent manner. The findings demonstrate the genetic importance of a lymphatic endothelial glycan (i.e., heparan sulfate) on cell traffic in the lymphatic microenvironment. Specifically, our findings focused on the lymphatic trafficking of carcinoma cells during lymph node metastasis. Exploring possible mechanisms in cell-based systems revealed unique and critical roles served by lymphatic endothelial HS in mediating lymphatic-directed tumor cell migration in response to the chemokine CCL21. This occurred through distinct roles served by two forms of lymphatic endothelial HS: (1) Lymphatic-secreted HS (presented on proteoglycans in lymphatic endothelial conditioned medium), which appeared to serve as a soluble co-receptor for CCL21-CCR7 association and migration signaling on tumor cells; and (2) Lymphatic cell-surface HS (displayed on lymphatic membrane-bound proteoglycans), which appears to concentrate CCL21 on the lymph endothelium, and thereby serves as a means by which lymphatic vascular chemokine gradients may be regulated in vivo.

### Unique role for heparan sulfate in the lymphatic microenvironment: Novel control of tumor migration by an important chemokine

As a result of unique sulfate modifications, HS has been identified as a mediator of chemokine binding in both cell-free preparations [26] and a limited number of cell biological contexts. In particular, HS has been found to play roles in chemokine-dependent inflammatory leukocyte extravasation [37] as well as the binding of inflammatory chemokines to ECM in clinical rheumatoid arthritis specimens [28]. To date, however, there are no data examining the genetic importance of HS in the lymphatic microenvironment and lymph node tumor-cell trafficking; and in carcinoma, the possible genetic importance of HS as a mediator of chemokine signaling and/or gradients has not been reported. While some recent data pointed to the importance of the CCL21-CCR7 axis during tumor-lymphatic chemo-attraction [8,9], we also noted that CCL21 is endowed with a C-terminus that is particularly rich in basic amino acids that may contribute to heparin binding [44], which we confirmed by heparin affinity chromatography using recombinant CCL21 (Figure 5A). With these considerations in mind, we found that pre-treatment of LEC with heparinase (to destroy cell-surface HS) inhibited murine Lewis lung carcinoma (LLC) invasion into the LEC-embedded collagen (Figure 1B), a process that was also CCL21 dependent. Blocking biosynthesis of HS by the LEC through siRNA resulted in similar effects in both murine and human lung carcinoma cells.

These insights led us to examine regional (inguinal) lymph node colonization by LLC tumor cells following experimental tumor establishment in syngeneic mice bearing a pan-endothelial alteration in HS biosynthesis. Initially, mutant (*Ndst1*^f/f^*TekCre*^+^) mice showed not only a reduction in LLC metastasis to regional lymph nodes, but also a reduction in CCL21 associated with metastatic tumor colonies (Figure 2B). Nodal metastasis in subcutaneous Lewis lung carcinoma models has been shown to occur via the afferent lymphatic conduit that intervenes between primary tumor and regional lymph nodes [45]. The findings support an important role for HS in mediating chemokine-dependent lymphatic-borne trafficking of tumor cells to the regional lymph nodes. However, this model may be somewhat limited by the fact that *Tek *drives pan-endothelial *Cre *activation; and we could not fully rule out the possibility that differences in regional lymph node colonization might be based on not only lymphatic *Ndst1 *deficiency, but also blood-endothelial *Ndst1 *deficiency. Nevertheless, the model was characterized by relatively heavy tumor metastasis to the regional lymph node, while very few/rare pan-keratin positive foci could be detected in the contralateral inguinal lymph node (or the more distant cervical lymph node; data not shown). This is consistent with the likelihood that lymph node metastases to regional lymph nodes occurred via intervening lymphatic vasculature. Moreover, the model was characterized by an absence of intra-tumor lymphatic vessels, and comparable lymphatic vessel densities in the peri-tumor tissue of mutant vs wildtype littermates. Thus, targeting HS sulfation in the lymph endothelium resulted in altered nodal metastasis in the mutant mice while the anatomic extent of the peri-tumoral lymphatic beds in mutant and wildtype littermates were comparable.

### Mechanistic considerations: Secreted and cell-surface bound heparan sulfate

How might one explain the marked alteration in nodal metastasis that was observed in the setting of the glycan-altered lymphatic endothelial conduit? Lymph node - colonizing tumor deposits in wildtype littermates were associated with abundant CCL21 that localized with the tumor deposits. However, CCL21 immunoreactivity associated with the limited tumor deposits found in mutant lymph nodes was reduced (Figure 2B, lower panels). Two possible mechanistic explanations that we explored involved alterations in chemokine-dependent tumor cell behavior that might result from mutation of either (1) HS secreted by the lymph endothelium, or (2) HS displayed on the lymphatic endothelial cell surface. We first hypothesized that genetic alteration of HS secreted by the lymphatic endothelium might disrupt the presentation of soluble CCL21 to CCR7 receptor (with altered downstream migration signaling) on the surface of metastasizing tumor cells, a phenomenon reminiscent of the ability of sulfated domains on HS to critically "cluster" heparin-binding chemokines (e.g., as in oligomerization of IL-8 or CCL2) for receptor presentation [32]. We also hypothesized that disrupting the sulfation of HS expressed on the lymphatic cell surface might alter gradient distributions of CCL21 tethered and concentrated on the HS chains. As in the case of vascular endothelial HS mediating MIP-2 gradients needed to drive trans-endothelial neutrophil migration [46], lymph endothelial HS might similarly mediate CCL21 gradients within lymph node lymphatic capillaries that in turn may facilitate the trafficking of tumor cells to the lymph node.

Mechanistic evidence for the first hypothesis (i.e., lymphatic-secreted HS serving as a chemokine co-receptor) was supported by initial cell-based studies wherein lymphatic endothelia were separated from tumor cells by lymphatic conditioned media (Figure 3A). Heparan sulfate is known to interact with basic amino acid motifs on several chemokines, and may serve as a co-receptor for the activation of certain chemokine receptors by specific chemokines. A known example is the requirement for HS during the activation of CXCR4 by CXCL12/SDF-1α [47]. However, evidence for the genetic importance of the glycan in mediating receptor binding and activation by such chemokines in biological systems is lacking. Given the chemokine- and lymphatic HS-dependent behavior of tumor cells demonstrated in the migration assays (Figure 3B, C), we focused on the CCL21/CCR7 axis, and asked whether genetic alteration of lymphatic-secreted HS might (*in-trans*) play a key role in mediating activation of migration signaling in the tumor cells. The results identified key roles for lymphatic HS secreted by the LEC into the conditioned medium on tumor-cell migration behavior. In addition, the presence of HS in the conditioned medium supported phosphorylation of both FAK as well as the migration-associated signaling intermediate GSK3β, a condition that was abolished by blockade of CCL21 in the conditioned medium or by inhibiting the biosynthesis of HS secreted into the conditioned medium (Figure 4). The latter was accomplished by siRNA-mediated targeting of HS chain initiation on lymphatic proteoglycans secreted into the medium (siXylT2) or by targeting HS chain sulfation (siNdst1) on lymphatic-secreted HS proteoglycans. Phosphorylation of the mitogen Erk1/2 in tumor cells in response to CM exposure, however, was not sensitive to siRNA mediated targeting of HS in the CM (Figure 4B), implying that the activation of chemokine-mediated migration-associated pathways (i.e., p-FAK, p- GSK3β) in the tumor cells appears to be more sensitive to the HS alteration than the effect on activation of a major mitogen (i.e., p-Erk1/2) pathway. Importantly, we questioned whether these signaling alterations on tumor cells that depend on the state of HS in the conditioned medium were associated with "upstream" alterations in the ability of CCL21 to associate with CCR7 G-protein coupled receptors on tumor cells. This appeared to be the case, as demonstrated through the use of proximity ligation assays using cultured tumor cells exposed to the intact versus HS-targeted conditioned media (Figure 5A, B). The findings were not trivially explained by a simple reduction in lymphatic chemokine production (i.e., in the conditioned medium) as a result of HS targeting in the LEC (Figure 5C). Rather, it appears that disrupting the biosynthesis of HS (which was also confirmed at the level of glycan sulfation for *Ndst1 *targeting; Figure 5D) may disrupt the presentation of HS-associated CCL21 chemokine to its receptor. This is reminiscent of solution-phase studies demonstrating critical oligomerization of some chemokines by glycosaminoglycans [26], and the findings thus demonstrate the genetic importance of the glycan as it serves (*in trans*) to effect chemokine-dependent tumor cell behavior in vitro and lymph node colonization in vivo.

Another mechanistic possibility is that disruption of HS expressed on the lymphatic endothelial surface may alter gradients of CCL21 concentrated on HS chains on the lymphatic endothelial surface and peri-lymphatic ECM of the lymph node. In the lymph node, in addition to expression in the high endothelial venules, CCL21 is expressed within the lymphatic endothelial-rich T-cell zones and inner lymph node cortex [14]. It is also expressed by the lymphatic endothelium of multiple other tissues. Heparan sulfate expressed on lymphatic membrane-bound as well as peri-lymphatic matrix proteoglycans might critically serve as a scaffold for tethering CCL21 gradients produced by lymphatic vessels. It should also be recognized that CCL21 may bind to other lymphatic endothelial proteins, such as podoplanin [48], which might also contribute to gradient formation and/or temper alterations in CCL21 gradients disrupted by targeting HS in vivo. Nevertheless, altering the biosynthesis of lymphatic HS may thus disrupt the ability of CCR7-expressing tumor cells to migrate/extravasate from the lymphatic vascular lumen of afferent lymphatic vessels (entering the lymph node) toward the chemokine-rich lymphatic vessel wall and surrounding matrix. While the latter anatomic region is not characterized by a basement membrane per se, it is a region wherein perlecan, a major secreted HS proteoglycan, has been identified [27]; and we also identified perlecan as the major secreted HS proteoglycan expressed by cultured primary lymphatic endothelial cells (Additional File 7 - Figure S7). Accordingly, any lumen-to-matrix flow of lymph entering the lymph node via afferent lymphatic vessels may support such a gradient, and trafficking CCR7-positive cells may extravasate and colonize the lymph node along the same pathway. In addition to HS on peri-lymphatic matrix proteoglycans, the findings in Figure 6 support the importance of lymphatic cell-surface bound HS proteoglycans in serving as an important source for CCL21 gradients. The absence of such gradients in the setting of HS disruption may thus limit the ability of the CCR7-expressing tumor cells to traffic out of afferent lymphatic vessels in the lymph node and colonize the lymph node parenchyma. Finally, it is also possible that integrins play a critical role (reviewed in [49]), working together with HS proteoglycans during cell adhesion and lymphatic transmigration events that contribute to this process.

### Re-visiting heparan sulfate in vivo: Lymph node metastasis in the setting of a lymphatic-specific heparan sulfate alteration

Our findings thus suggested at least two mechanisms whereby lymphatic HS might be specifically required for CCL21-mediated colonization of lymph nodes by tumor cells: Namely, through the ability of lymphatic endothelial HS to as serve as either a co-receptor for chemokine-mediated tumor migration signaling or as an essential scaffold for peri-lymphatic chemokine gradients (or both). With these mechanisms in mind, the in vivo effects of a pan-endothelial deficiency in HS sulfation on regional lymph node metastasis (Figure 2) suggested that a lymphatic-exclusive mutation in *Ndst1 *might also disrupt regional lymph node metastasis using the same tumor model. This was achieved through the use of a mouse construct wherein *Cre *was driven by the lymphatic-endothelial specific promoter *Prox1 *[50]; with the added advantage that conditional mutants could be induced to develop the lymphatic-specific alteration in *Ndst1 *(through administration of tamoxifen to activate *Prox1 *driven *Cre*) exclusively in lymphatic vessels immediately prior to the experiment. Mice therefore did not develop with a tissue-targeted *Ndst1 *deficiency (as they would have in the *TekCre *model), and we applied a tamoxifen induction schedule that resulted in efficient *Cre *activation in lymphatic vessels, including those in lymph nodes (Figure 7A). Using this model, a marked reduction in lymph node metastasis was noted in *Ndst1*^f/f^*Prox1*^*+/CreERT2 *^mutants (Figure 7C). Importantly, in examining CCL21 immunoreactivity in the lymph nodes of *Ndst1*^f/f^*Prox1*^*+/CreERT2 *^mutants, while the signal localized to regions of tumor deposits, the relative expression of CCL21 in those regions was markedly reduced (Figure 7D, bottom panels) as compared to that in the lymph nodes of *Cre *negative control littermates (Figure 7D, upper panels), where tumor deposits were essentially fully decorated with CCL21. These findings parallel our in vitro results that examine lymphatic-HS dependent tumor-cell binding and responses to CCL21 (Figures 3, 4, 5), and support an important role for HS produced by the lymphatic endothelium in mediating the binding of CCL21 to node-metastatic tumor cells in vivo.

In addition to considering limitations in the applicability of our findings, it is also worthwhile to re-examine our current knowledge about the nature of vascular endothelial disorders involving altered *Ndst1 *expression. First of all, while the above proof-of-principle studies were carried out using both mouse as well as human lung carcinoma cell lines, we cannot over-generalize our findings and mechanisitic insights to lymph node metastasis from all primary lung carcinomas. Variation might occur in relation to CCL21 distribution/expression, the degree of CCR7 over-expression by specific tumors, or even the degree of HS expression in distinct lymphatic vascular beds. Although this is the first study that specifically targets HS expression in the lymphatic endothelium, we have gained some insights from other work that has focused on vascular disorders associated with alterations in vascular *Ndst1 *expression. It is noteworthy, for example, that experimental blood-endothelial as well as vascular smooth muscle - targeted alterations in *Ndst1 *expression may result in marked alterations in chemokine-mediated inflammatory cell infiltration in the setting of transplant [51] as well as altered growth factor dependent vascular proliferation responses [52]. Further work is necessary to characterize alterations in lymphatic endothelial *Ndst1 *expression in human lymphatic disease. It is possible, from the findings herein, that variations in expression might result in altered carcinoma cell trafficking patterns.

## Conclusions

**Figure 8:**
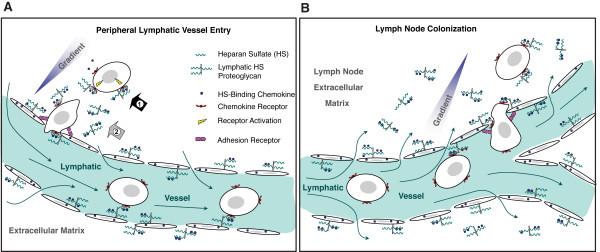
**Summary of mechanistic considerations: Role of lymphatic endothelial heparan sulfate in chemokine-mediated lymph node metastasis**. **A**. In peripheral lymphatic vessels (e.g., lymphatic vascular bed of a primary tumor), HS-binding chemokines produced by the lymphatic endothelium such as CCL21 may "cluster" on lymphatic-bound as well as secreted HS proteoglycans. In the setting of intra-lymphatic flow (thin arrows), spatial gradients of the chemokine scaffolded on HS chains in the extracellular matrix and lymphatic endothelium may facilitate tumor-cell migration into lymphatic vessels. This may occur in coordination with other adhesion systems such as integrins or selectins. Clustering of chemokines (as dimers in this example) on HS in the peri-lymphatic extracellular matrix may also be critical for receptor activation (e.g., CCR7) on migrating tumor cells. Targeting HS biosynthesis may thus alter chemokine-receptor interactions (large black arrow, mechanism "1") as well as chemokine gradients (large grey arrow, mechanism "2"). **B**. In the lymph node, similar principles may apply; however, the direction of trans-lymphatic flow and chemokine gradient are reversed, facilitating extra-vasation (colonization) of trafficking tumor cells expressing cognate chemokine receptors. Interfering with the biosynthesis of lymphatic heparan sulfate (e.g., genetically targeting the sulfation of HS through lymphatic endothelial *Ndst1 *mutation) may thus abrogate lymph node metastasis by altering such co-receptor and gradient-mediating functions served by the glycan.

These findings support important roles for lymphatic HS in mediating both the ability of tumor cells to colonize lymph nodes and the ability of chemokine to bind and effect the migration of tumor cells in a receptor-dependent manner. Figure 8 summarizes the mechanisms whereby heparan sulfate produced by the lymphatic endothelium may critically mediate chemokine-dependent lymph node trafficking by tumor cells. The mechanisms, involving co-receptor as well as gradient-scaffolding functions by the glycan, may apply to both invasion of lymphatic vessels in the primary tumor (Figure 8A) as well as lymphatic extravasation and colonization by lymph-borne tumor cells entering regional lymph nodes (Figure 8B). It is important to recognize that while we have demonstrated how this applies to tumor cell invasion (including migration signaling) by a prototypical heparin-binding chemokine (CCL21), the mechanisms may also apply collectively to other HS-binding chemokines (e.g., CXCL12, among others) that may function in redundant manners to drive lymphatic tumor cell traffic. Further studies are needed to weigh the relative impact of HS-mediated chemokine gradients and/or co-receptor functions on migrating tumor cells in distinct tumor models in vivo. Nevertheless, the genetic proof-of-principle herein suggests that targeting HS in the lymphatic system may have significant effects in altering tumor-lymphatic cell traffic as well as lymph node metastasis in clinical cancer.

## Methods

### Cell culture and treatments

Primary human lung lymphatic endothelial (hLEC) cells (Lonza) were cultured in fully supplemented EBM2 endothelial basal medium (with EGM2 bullet kit; Lonza). Cells were determined to be >99% pure at the 3^rd ^passage by nuclear staining for Prox-1 via immunofluorescence. Lewis lung carcinoma (LLC) cells (ATCC) and stable GFP-expressing LLC cells were cultured in DMEM (Invitrogen) supplemented with 10% FBS (Atlanta Biologicals). Human adenocarcinoma cells H1650 (ATCC) were cultured in RPMI-1640 (Invitrogen) supplemented with 10% FBS. All siRNA duplexes were from IDT (Coralville, IA), and transfected into cells according to manufacturer instructions. For heparinase treatment, cells were incubated with heparinases I, II and III (kindly provided by Dr. J.D. Esko) in serum-free EBM2 medium at 37°C, 5% CO_2 _for 4 h. For heparin wash, the cells were washed with PBS, incubated in 100 μg/mL heparin (Sigma) in PBS at room temperature for 10 min with gentle shaking and washed with PBS twice to remove any residual heparin. To block specific chemokine or chemokine receptor signaling, cells were incubated with α-CCL21 (1:100), α-CCL5 (1:100) or α-CCR7 (1:100, R&D) at 37°C, 5% CO_2 _for 30 min (for harvesting CM) to overnight (for transwell migration assay).

### Modified transwell migration assays

To assess the mobility of tumor cells toward hLEC, a modified transwell migration assay was performed in two ways that differed in the manner hLEC were plated. For collagen matrix-based invasion studies, 1 × 10^5 ^hLEC were embedded into 100 μL of type I collagen gel containing 2.7 mg/mL PureCol (Advanced Biomatrix, San Diego, CA) in 1X DMEM (pH 7.3), applied as a liquid to cover the lower side of transwell inserts (5.0 μm in pore size, Corning), and incubated at 37°C, 5% CO_2 _for six hours. Inserts were then inverted and placed into 24-well plates (500 μL/well) containing pre-warmed basal (i.e., growth factor free, un-supplemented) EBM2 with 2% FBS. For migration toward conditioned medium (CM) overlying monolayer-cultured hLEC, 5 × 10^4^/well hLEC were seeded directly into 24-well plate, and treated accordingly. For both assays, 1 × 10^5 ^H1650 cells pre-labeled with Calcein AM (following the manufacturer instructions; Invitrogen) or stable GFP-expressing LLC cells were resuspended in 100 μL of EBM2 with 2% FBS and loaded on top of the insert. The migration proceeded in a 37°C, 5% CO_2 _incubator for overnight. Inserts were then treated with either 0.2% type I collagenase (Sigma) at 37°C for 1 h (for collagen matrix-based migration assay) or trypsin-EDTA (Invitrogen) for 5 min (for chemotaxis migration assay) with gentle rocking. Digests (including media from lower wells) were collected, transferred to eppendorf tubes, and centrifuged at 5,000 × g for 5 min. Cell pellets were resuspended in 15 μL PBS, with 1.5 μL samples loaded onto a 96-well Terasaki plate (Robbins Scientific), and imaged under a fluorescence microscope (Nikon Eclipse 80i). Images were taken under 40× magnification and analyzed with NIH Image J software. All assays were set up in triplicate with at least three independent experiments performed for each assay. In a subset of chemotaxis migration assays, purified HS isolated from the CM of control hLEC was dosed into the lower wells of siXylT2 targeted hLEC. To assess dose-response, fractions of the total HS needed to achieve full rescue of invasion (i.e., to a degree comparable to that achieved toward siDS control hLEC) by the tumor cells were added/tested in separate wells containing siXylT2 targeted hLEC in the lower wells. Isolation/purification of HS from the LEC was carried out according to the method of Bame, et al. [53].

### Reverse-transcription followed by quantitative real-time PCR (RT-qPCR)

Total RNA was extracted from cells using RNAqueous 4-PCR kit (Ambion) and reverse transcribed into cDNA with SuperScript III kit (Invitrogen) according to the manufacturers' instructions. Real-time PCR was performed with iQ Sybr Green Supermix Kit (BioRad). The primer sequences (5' to 3') used for real-time PCR were as follows: human Ndst1 forward, GGACATCTGGTCTAAG, and reverse, GATGCCTTTGTGATAG; human XylT2 forward ACGTTCAACCGCAAACTACC, and reverse, ATTGCTCAGTTCCCCATCTG; human CCL21 forward, GCCTTGCCACACTCTTTCTC, and reverse, CAAGGAAGAGGTGGGGTGTA; human CCR7 forward, TTCTTCACTGTCCTCCAAGC, and reverse, ACATTTCCCTTGTCCTCTCC. The PCR condition was 95°C for 3 min followed by 40 cycles of 95°C for 30 sec, 59°C for 30 sec and 72°C for 30 sec. Relative expression of target gene against β-actin was calculated using 2^-ΔΔCt ^method [54]. Primers used for quantitative PCR of major HS proteoglycan core proteins produced by hLEC (Additional File 7 - Figure S7) are described in Additional File 8 - Table S1.

### Preparation of conditioned medium (CM) from hLEC

To harvest conditioned medium (CM) for Proximity Ligation Assays (PLA; Duolink) and for HS isolation, hLEC were treated accordingly, washed with PBS, and incubated in basal EBM2 containing 5% diethylaminoethyl (DEAE) anion-exchange treated FBS (glycosaminoglycan-free) for 24 h. To harvest CM for stimulating LLC cells in all other assays, hLEC were incubated in basal EBM2 medium alone for 5 h. The supernatant was then collected and briefly centrifuged to remove the cell debris.

### Purification of HS from hLEC conditioned medium and characterization HS disaccharides

HS was isolated from the CM of hLEC cells as previously described [53], and digested into disaccharides via enzymatic depolymerization with heparinase (2 IU each of heparin lyases I,II,III; IBEX) overnight at 37°C in 50 μl buffer (40 mM ammonium acetate and 3.3 mM calcium acetate, pH 7). (For some cell-based studies, purified HS was used intact (i.e., undigested), and dosed into the cell medium.) HS disaccharide analysis was carried out using quantitative liquid chromatography/mass *spectrometry *according to published methods [55]. Briefly, HS digests were dried down, and to each sample the following were added: [^12^C_6_]aniline (15 μl, 165 μmol) and 15 μl of 1 M NaCNBH_3 _(Sigma/Aldrich) freshly prepared in dimethylsulfoxide: acetic acid (7:3, vol/vol). Aniline labeling of the reducing ends of disaccharides was carried out at 37°C for 16 hr and products were dried down. The derivatized disaccharides were separated on a C18 reversed phase column (0.46 × 25 cm; Vydac) with ion pairing agent (dibutylamine, Sigma/Aldrich) [56]. Eluted ions of interest were monitored in negative ion mode (on a classic quadrupole ion trap mass spectrometer; Thermo-Finnigan). The capillary temperature and spray voltage were maintained at 140°C and 4.75 kV, respectively. Accumulative extracted ion current was computed, and data were analyzed (Qual Browser software; Thermo).

### Western immunoblots

Cells were lysed in RIPA buffer (Teknova) supplemented with protease inhibitor cocktails (Sigma) and phosphatase inhibitor cocktails (Santa Cruz). The protein concentration was determined with Protein assay dye reagent (BioRad). A total 50 to 70 μg protein was separated on a 4%-20% gradient gel (BioRad), electrotransferred to a nitrocellulose membrane, probed with one of the following primary antibodies: CCL21 (1:1000, R&D), FAK (1:100), p-FAK (Tyr397, 1:1000) (Santa Cruz, CA), p-GSK3β (Ser9, 1:1000) (Enzo Life Sciences), GSK3β (1:1000, Upstate), Tubulin (1:10,000, Sigma) and Ndst1 (1:1000, Abcam). Additional primary antibodies included anti-CCR7 (1:5000, Novus) and anti-β-actin (1:5000, Sigma). After two washes with Tris-buffered saline/0.1% Tween-20 (TBST), the membrane was incubated with an HRP-conjugated secondary antibody and visualized using SuperSignal chemiluminescent substrate (Pierce). The signal density was quantified using Adobe Photoshop 7.0.

### Detection of CCL21 in conditioned medium (CM) from hLEC

To measure the secreted CCL21 by hLEC, 3 × 10^5 ^hLEC cells were seeded onto wells of a 6-well plate, and treated accordingly (i.e., siRNA/transfection treatments targeting HS biosynthesis and appropriate control transfections). Cells were washed with PBS once and basal EBM2+5% FBS was added to the cells. A transwell insert (0.4 μm in pore size, Corning, Cat. #3412) was placed into each well and 100 μL Heparin sepharose (GE Healthcare, Piscataway, NJ) pre-washed with PBS and resuspended in 1 mL EBM2/5% FBS was loaded on top of the insert. The cells were incubated in 37°C, 5% CO_2 _incubator for 24 h. Following the incubation, the transwell insert was transferred to a clean 6-well plate and Heparin sepharose was collected with PBS wash until no sepharose beads were visible on the insert. All washes were pooled and briefly centrifuged with supernatant carefully removed. 2X protein loading buffer and β-mercaptoethanol were added to the sepharose beads to a final concentration of 1 × and 5% respectively. The samples were boiled at 95°C for 5 min, loaded onto a SDS-PAGE gel, and CCL21 was detected by Western immunoblot.

### Immunofluorescence

hLEC growing on glass coverslips were treated accordingly, fixed in 4% paraformaldehyde, blocked with PBS/1%BSA at room temperature for 1 h and stained with anti-CCL21, anti-CCR7 (R&D, 1:100) or isotype-matched control IgG (1:100, Vector Lab) at 4°C overnight. After PBS wash several times, the cells were incubated with Biotinylated secondary antibody (1:500, Vector Lab) at room temperature for 1 h followed by Alexa 488-conjugated streptavidin (2 μg/mL, Invitrogen) and mounted with VectaShield with DAPI (Vector Labs). All images were taken under a fluorescence microscope (Nikon Eclipse 80i; 100× or 400×).

### Heparin affinity chromatography

Heparin affinity chromatography was used to assess the binding of CCL21 and bFGF to column-immobilized heparin. Briefly, 100 μL Heparin sepharose was loaded onto chromatography column (BioRad) and equilibrated in wash buffer (0.15 M NaCl in 25 mM HEPES, pH 7.2). Human CCL21 or bFGF (2 μg, Peprotech) was then added to the column in 100 μL wash buffer. Stepwise elutions (200 μL each) with wash buffer containing increasing [NaCl] (up to 3 M maximum) were then applied, and each elution was collected for subsequent protein analysis. 10% of each elution was prepared in SDS sample buffer with 5% β-mercaptoethanol, boiled, and applied to a 4%-20% gradient SDS-PAGE gel for electrophoresis. Gels were silver-stained (Pierce) and photographed.

### Duolink assays

The specific interaction between CCL21 and CCR7 was determined using a proximity ligation assay (PLA; Duolink, Olink Bioscience) following manufacturer instructions with the following primary antibodies used: anti-CCL21 (R&D, MAB3661, 1:100) and anti-CCR7 (Novus, 1:250).

### Animal Models and Tumor Metastasis Studies

All animal experiments were reviewed and approved by the Institutional Animal Care and Use Committee (IACUC). All mice used in the studies were between four to eight weeks of age. Mutant *Ndst1*^f/f^*TekCre*+ and *Ndst1*^f/f^*TekCre*- littermate control mice on the C57Bl/6 background were generated as previously described [37]. Mutant *Ndst1*^f/f^*Prox1*^*+/CreERT2 *^mice (and *Ndst1*^f/f^*Prox1*^*-/CreERT2 *^control littermates) were generated through breeding of the *Prox1*^*+/CreERT2 *^construct (generously provided by Dr. Guillermo Oliver at St. Jude Children's Research Hospital, Memphis, TN) [43] with *Ndst1*^f/f ^conditional mutants (first described in [37]), following extensive backcrossing of both lines onto the C57Bl/6 background. To evaluate the activity of *Cre *recombinase, *Prox1*^*+/CreERT2 *^mice were also bred to *Rosa26R *reporter mice (Jackson Laboratory). To induce *Cre *recombinase activity, tamoxifen (Sigma) dissolved in corn oil was intraperitoneally (i.p.) injected into all mice (i.e., both *Prox1*^*+/CreERT2 *^and *Prox1*^*-/CreERT2 *^controls) at 0.12 mg/g body weight for five consecutive days. All mice were maintained in a pathogen-free facility on a 12 h:12 h ligh-dark cycle with food and water provided *ad libitum*. To investigate metastasis of tumor cells to regional lymph node, mice were anaesthetized with isoflurane gas vapor and 2.5 × 10^5 ^LLC were subcutaneously (s.c.) injected into a skin fold in the caudal-medal aspect of the inguinal region. For experimental mice (undergoing tamoxifen injection), LLC tumor cells were administered on Day 7 following the last injection of tamoxifen. Fourteen days after LLC injection, mice were sacrificed with both primary tumor and left subiliac LN isolated. The length (L) and width (W) of the primary tumor were measured with caliper and the tumor size was calculated as 0.5 × L × W^2^. (During this time period for growth, injection of GFP-expressing LLC tumor cells resulted in subcutaneous tumors that grew to ~0.5 cm diameter, and were comparable in both mutant and wildtype mice. Larger tumors were avoided in the *TekCre *model, given known effects of the mutation on the subcutaneous growth and vascularization of larger LLC tumors [25].) Isolated tissues were fixed in 10% buffered neutral formalin (Fisher) at room temperature for 24 h.

Purification of LEC from *Ndst1*^f/f^*TekCre*^*+ *^mutant mice (and *Cre*^- ^wildtype littermates) for analyses of Ndst1 expression were carried out using LEC isolated from two tissue sources: (1) LEC isolation from freshly resected mouse lungs, wherein lungs were washed, digested in 0.2% collagenase (Sigma) following mechanical disruption with scissors, strained (40 μm strainer) for cell enrichment, labeled with biotinylated antibody to mouse LYVE-1, and purified into culture (EBM-2 growth medium; Lonza) using anti-biotin magnetic beads according to manufacturer (Miltenyi) instructions; (2) LEC isolated from lymphatic proliferative lesions (abdominal oil granuloma/lymphangioma lesions) following the method of Mancardi and others [57].

### Whole-mount LacZ staining

Whole-mount lacZ staining was performed as previously described [58]. Briefly, LN and other tissues were freshly isolated, fixed in β-galactosidase fixative (0.2% glutaraldehyde, 5 mM EDTA and 2 mM MgCl_2 _in PBS) at room temperature for 2 h, washed three times (30 min each wash) in β-galactosidase wash buffer (0.01% sodium deoxycholate, 0.02% NP-40 and 2 mM MgCl_2 _in PBS), and stained overnight in β-galactosidase staining solution (1 mg/mL X-gal, 5 mM K_4_Fe(CN)_6 _and 5 mM K_3_Fe(CN)_6 _in β-galactosidase wash buffer) at room temperature with gentle rocking. For histological examination, stained tissues were washed three times in PBS and post-fixed in 10% buffered neutral formalin at 4°C overnight, embedded into paraffin and 5-μm sections were cut.

### Immunohistochemistry

Formalin-fixed tissues were embedded into paraffin and 5-μm thick sections were made. Following depraffinization and rehydration through a decreasing ethanol series, the section slides were cooked in antigen retrieval buffer (10 mM sodium citrate, pH 6.0) under high pressure for 10 min. The endogenous peroxidase activity was blocked with 3% hydrogen peroxide in dH_2_O for 10 min and the endogenous biotin blocked with Avidin-Biotin kit (Vector Lab) based on manufacturer instructions. A third blocking with 5% normal horse serum in TBST was performed at room temperature for 1 h. Tissue sections were then incubated with primary antibody at 4°C overnight. The following antibodies were used: α-LYVE1 (1:2000, R&D), α-CCL21 (1:2000, R&D), α-pan-keratin (1:5000, Cell Signaling). All antibodies were tested for conferring specific staining with isotype-matched IgG as negative control in preliminary experiments. Then the tissue sections were stained with a biotinylated secondary antibody (1:1000, Vector Lab) with the signal amplified and developed using Vectastain ABC kit followed by either DAB or Vector Blue substrate (Vector Lab). To quantify the pan-keratin and CCL21 signals within the lymph node, two sections separated by 100 μm were imaged under 40× magnification. The positive signals were quantified using NIH image J software and averaged to represent the signal within each lymph node.

### Heparin binding ELISA

Heparin-binding ELISA was performed using a heparan sulfate binding plate (BD Biosciences) following the manufacturer's instructions. Briefly, the 96-well plate was coated with 25 μg/mL porcine intestinal heparan sulfate (Sigma) in PBS overnight at room temperature. After washing with PBS containing 0.05% (v/v) Tween 20 and blocking with 0.2% (w/v) gelatin in PBS, wells were incubated for 2 h with recombinant human CCL21 (Peprotech) serially diluted in PBS. Wells were then washed, and biotinylated anti-CCL21 polyclonal antibody was added at 200 ng/mL in blocking buffer. After washing, Streptavidin-HRP (R&D) was added (1:200 in blocking buffer for 30 min) followed by PBS wash. Tween, substrate solution (1:1 H_2_O_2 _: tetramethylbenzidine) was added and absorbances were read at O.D. 650 nm after 10-min incubation at room temperature.

### Statistical analyses

Quantitative data were presented as mean ± SD for three replicates or independent experiments. Significance between groups was calculated with two-tailed Student's *t *test. Difference of lymph node metastasis between *Ndst1*^f/f^*TekCre*+ and *Ndst1*^f/f^*TekCre*- or between *Ndst1*^f/f^*Prox1*^*+/CreERT2 *^and *Ndst1*^f/f^*Prox1*^*-/CreERT2 *^mice was performed using the Rank Order test. A value of *P *< 0.05 was considered statistically significant.

## Competing interests

The authors declare that they have no competing interests.

## Authors' contributions

XY carried out and designed modified chemotaxis assays, siRNA and glycan chemical targeting strategies, genetic targeting and tumor models in vivo, and drafted the manuscript. JT participated in experiment design for collagen-based tumor-lymphatic invasion studies. RL participated in glycan analysis, including liquid chromatography/mass spectrometry studies. SCJ assisted with siRNA, immunohistology, and proximity ligation assays. RSS assisted with the *Prox1*^*+/CreERT2 *^mouse construct and tamoxifen induction trials for adult mouse tumor studies. TMH participated in methodology to detect chemokine in conditioned media, and assisted with intellectual content on HS-chemokine interactions. MMF conceived of the study, facilitated and designed experiments, and helped draft the manuscript. All authors read and approved the final manuscript.

## Supplementary Material

Additional file 1 **Figure S1: siRNA transfection knocks down the expression of HS biosynthetic enzymes but not CCL21 or CCR7 in human lymphatic endothelia**. **A**. Primary human lymphatic endothelial cells (hLEC) were transfected with either control RNA (siDS), siNdst1 or siXylT2. The steady-state mRNA levels of Ndst1, XylT2, CCL21 or CCR7 were examined by RT-qPCR and indexed to that of β-actin. The target/β-actin expression ratio in siDS-transfected hLEC was arbitrarily defined as 1. **B**. hLEC were transfected with either control scrambled-duplex RNA (siDS) or siNdst1. The protein level of Ndst1 was determined by Western immunoblot. Tubulin was measured as a loading control. Click here for file

Additional file 2 **Figure S2: siNdst1 transfection alters the sulfation status of lymphatic-secreted heparan sulfate**. HS was purified from the conditioned medium of hLEÇ transfected with control scrambled-duplex RNA (siDS) or siNdst1, and the sulfation status was examined by disaccharide analysis using liquid chromatography/mass spectrometry. Disaccharides listed below axis are named according to published nomenclature (reference #55 from main REFERENCES section). Click here for file

Additional file 3 **Figure S3: CCL21 binding to plate-immobilized heparin**: Increasing concentrations of recombinant human CCL21 were exposed to porcine intestinal HS pre-bound to an HS-binding plate, followed by washing and measurement of bound chemokine through ELISA to assess solid-phase binding of the chemokine to plate-immobilized glycan. (Estimated Kd ~ 100 ng/ml or approximately 8.3 nM.) Click here for file

Additional file 4 **Figure S4: CCR7 is expressed on human lymphatic endothelial cells**: **A**. The expression of CCR7 on the surface of hLEC was detected using anti-CCR7 antibody (green, left panel) by immunofluorescence. As a negative control, isotype-matched IgG (green, right panel) was used. The nuclei were stained with DAPI (blue). Images were taken under 100× magnification. Scale bar, 100 μm. **B**. Lysates from cultured hLEC were assayed for CCR7 expression by Western immunoblot (right lane), with expression in cultured LLC tumor cells (left lane) as a reference, and β-actin expression shown below as a loading control. Click here for file

Additional file 5 **Figure S5: Lymphatic vascular proliferation in lymph nodes of tumor-bearing mutant mice**: Lymphatic vascular density was plotted for *Ndst1*f/f *Prox1*+/*Cre*ERT2 mutant mice versus that of *Cre*-negative control littermates by quantifying LYVE-1 signal per lymph node (LN) (in arbitrary signal/LN units, shown in scatter-plot on the left). Graph on the right shows mean values (+/-SD) for an experiment examining N = 6 mutants versus N = 5 control mice. *P *= 0.14 for the difference in mean values. Click here for file

Additional file 6 **Figure S6: Immunolocalization of CCL21 and LYVE-1 in regional lymph nodes from tumor-bearing mice**: Lymph node sections from wildtype LLC tumor-bearing mice were immuno-stained with LYVE-1 (brown) and CCL21 (blue). Numerous CCL21+ cells (which co-localized with pan-keratin positive tumor deposits; see Figure 7D) were found in lymphatic-vascular rich regions of the lymph node. In addition, in some regions of lymphatic vessles, areas of co-localization of CCL21 with LYVE-1 (pink arrows) were noted (Bar = 50 μm). Click here for file

Additional file 7 **Figure S7: Spectrum of major heparan sulfate proteoglycan core proteins expressed by human lymphatic endothelial cells**: The steady-state mRNA levels of indicated HS proteoglycan core proteins were examined in hLEC by RT-qPCR and presented as percentage expression relative to that of β-actin. Plot shows secreted as well as cell-surface bound core proteins. Click here for file

Additional file 8 **Table S1: Primer Sequences (Forward/Reverse) used for Quantitative PCR**. of major human HS core proteins expressed by primary human lymphatic endothelial cells. Click here for file
