# Supplementary figures and images for: A critical role for lymphatic endothelial heparan sulfate in lymph node metastasis
Source: Mol Cancer. 2010 Dec 20;9:316. doi: 10.1186/1476-4598-9-316 (PMC3019167; doi:10.1186/1476-4598-9-316)

**A**

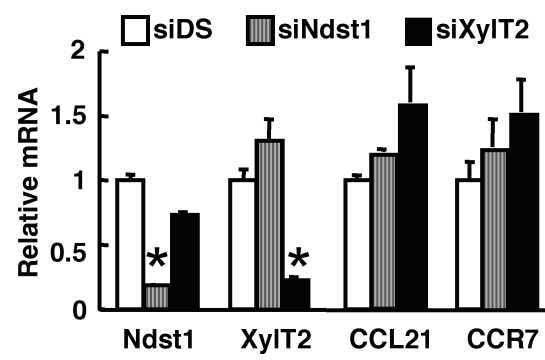

**B**

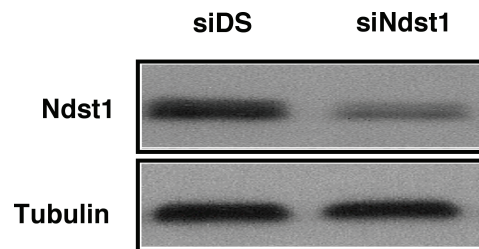

Supplement: Additional file 1 — Figure S1: siRNA transfection knocks down the expression of HS biosynthetic enzymes but not CCL21 or CCR7 in human lymphatic endothelia. A. Primary human lymphatic endothelial cells (hLEC) were transfected with either control RNA (siDS), siNdst1 or siXylT2. The steady-state mRNA levels of Ndst1, XylT2, CCL21 or CCR7 were examined by RT-qPCR and indexed to that of β-actin. The target/β-actin expression ratio in siDS-transfected hLEC was arbitrarily defined as 1. B. hLEC were transfected with either control scrambled-duplex RNA (siDS) or siNdst1. The protein level of Ndst1 was determined by Western immunoblot. Tubulin was measured as a loading control. [file 1476-4598-9-316-S1.PDF]

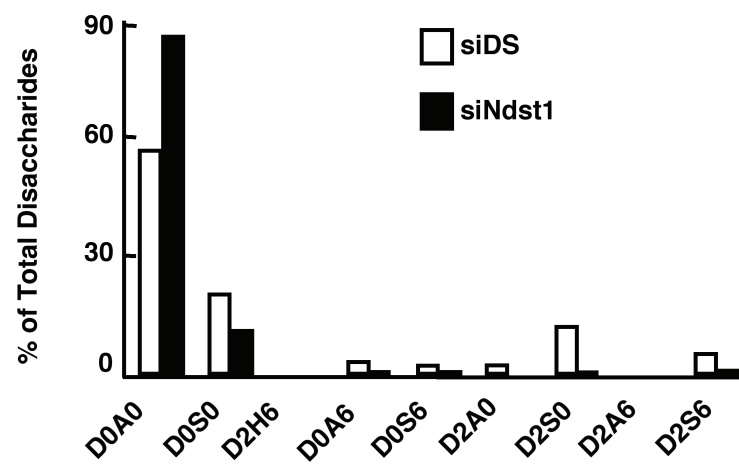

Supplement: Additional file 2 — Figure S2: siNdst1 transfection alters the sulfation status of lymphatic-secreted heparan sulfate. HS was purified from the conditioned medium of hLEÇ transfected with control scrambled-duplex RNA (siDS) or siNdst1, and the sulfation status was examined by disaccharide analysis using liquid chromatography/mass spectrometry. Disaccharides listed below axis are named according to published nomenclature (reference #55 from main REFERENCES section). [file 1476-4598-9-316-S2.PDF]

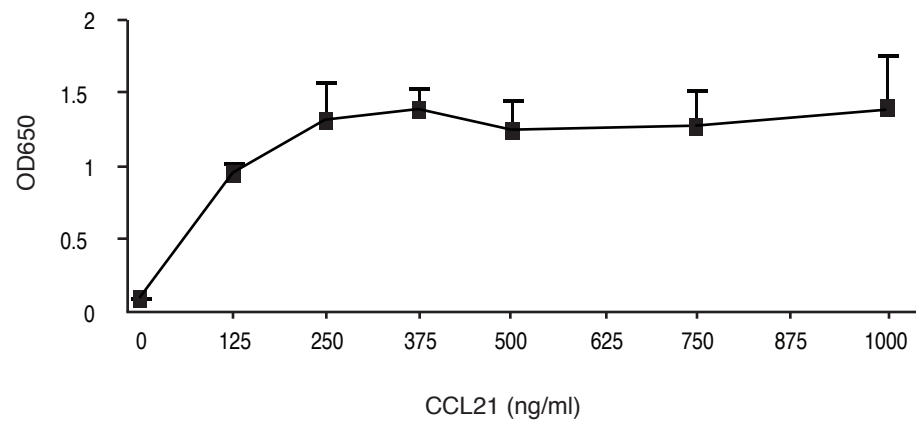

Supplement: Additional file 3 — Figure S3: CCL21 binding to plate-immobilized heparin: Increasing concentrations of recombinant human CCL21 were exposed to porcine intestinal HS pre-bound to an HS-binding plate, followed by washing and measurement of bound chemokine through ELISA to assess solid-phase binding of the chemokine to plate-immobilized glycan. (Estimated Kd ~ 100 ng/ml or approximately 8.3 nM.) [file 1476-4598-9-316-S3.PDF]

**A**

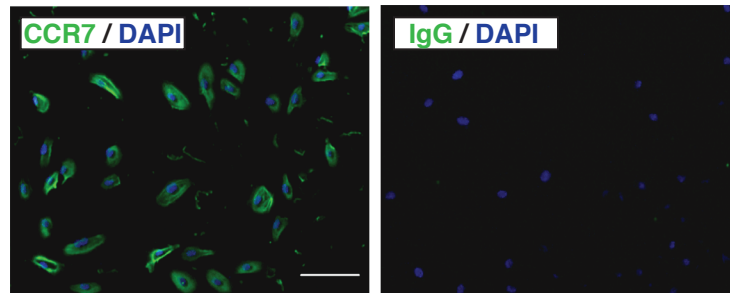

**B**

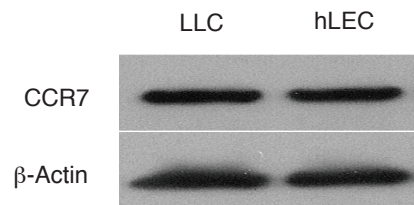

Supplement: Additional file 4 — Figure S4: CCR7 is expressed on human lymphatic endothelial cells: A. The expression of CCR7 on the surface of hLEC was detected using anti-CCR7 antibody (green, left panel) by immunofluorescence. As a negative control, isotype-matched IgG (green, right panel) was used. The nuclei were stained with DAPI (blue). Images were taken under 100× magnification. Scale bar, 100 μm. B. Lysates from cultured hLEC were assayed for CCR7 expression by Western immunoblot (right lane), with expression in cultured LLC tumor cells (left lane) as a reference, and β-actin expression shown below as a loading control. [file 1476-4598-9-316-S4.PDF]

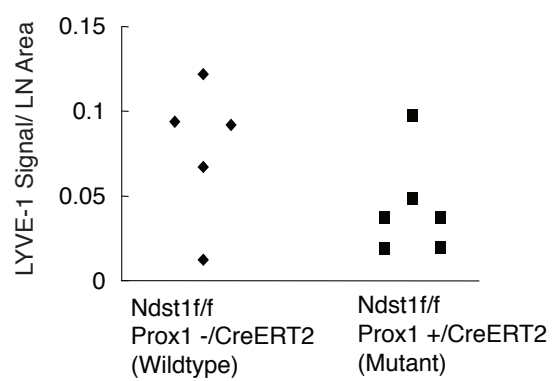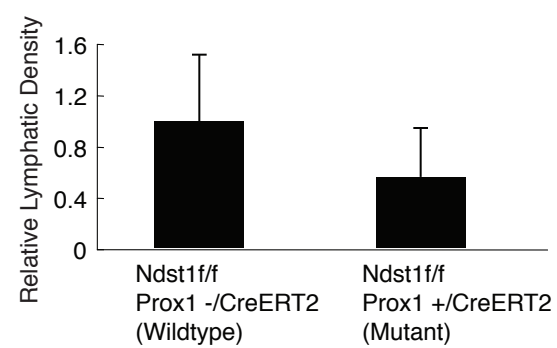

Supplement: Additional file 5 — Figure S5: Lymphatic vascular proliferation in lymph nodes of tumor-bearing mutant mice: Lymphatic vascular density was plotted for Ndst1f/f Prox1+/CreERT2 mutant mice versus that of Cre-negative control littermates by quantifying LYVE-1 signal per lymph node (LN) (in arbitrary signal/LN units, shown in scatter-plot on the left). Graph on the right shows mean values (+/-SD) for an experiment examining N = 6 mutants versus N = 5 control mice. P = 0.14 for the difference in mean values. [file 1476-4598-9-316-S5.PDF]

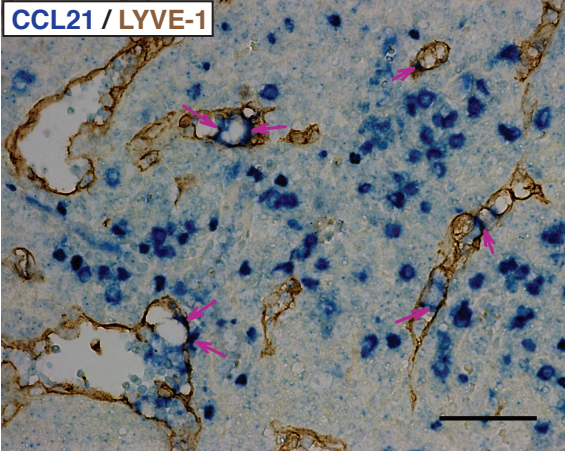

Supplement: Additional file 6 — Figure S6: Immunolocalization of CCL21 and LYVE-1 in regional lymph nodes from tumor-bearing mice: Lymph node sections from wildtype LLC tumor-bearing mice were immuno-stained with LYVE-1 (brown) and CCL21 (blue). Numerous CCL21+ cells (which co-localized with pan-keratin positive tumor deposits; see Figure 7D) were found in lymphatic-vascular rich regions of the lymph node. In addition, in some regions of lymphatic vessles, areas of co-localization of CCL21 with LYVE-1 (pink arrows) were noted (Bar = 50 μm). [file 1476-4598-9-316-S6.PDF]

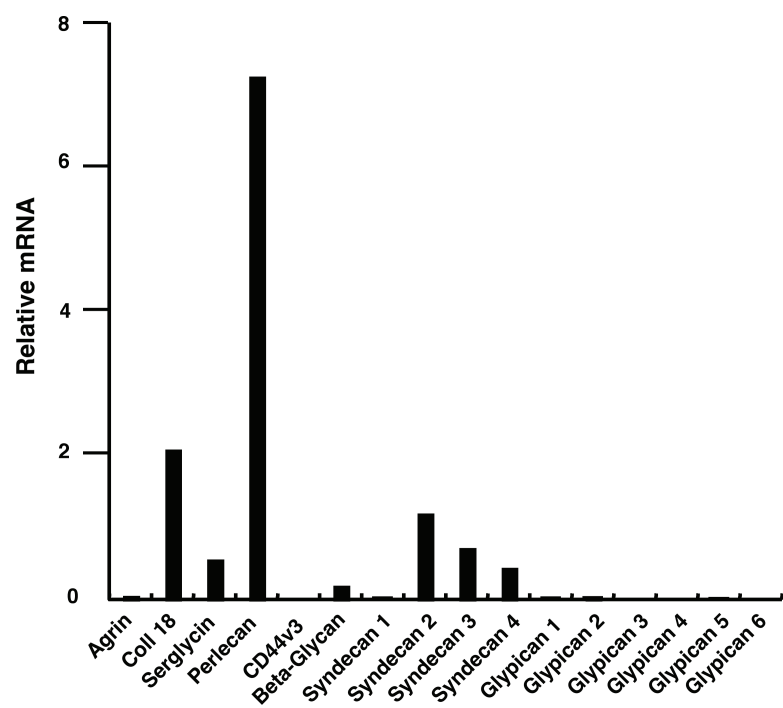

Supplement: Additional file 7 — Figure S7: Spectrum of major heparan sulfate proteoglycan core proteins expressed by human lymphatic endothelial cells: The steady-state mRNA levels of indicated HS proteoglycan core proteins were examined in hLEC by RT-qPCR and presented as percentage expression relative to that of β-actin. Plot shows secreted as well as cell-surface bound core proteins. [file 1476-4598-9-316-S7.PDF]
