# Supplementary material for: A critical role for lymphatic endothelial heparan sulfate in lymph node metastasis
Source: Mol Cancer. 2010 Dec 20;9:316. doi: 10.1186/1476-4598-9-316 (PMC3019167; doi:10.1186/1476-4598-9-316)
Supplement: Additional file 8 — Table S1: Primer Sequences (Forward/Reverse) used for Quantitative PCR. of major human HS core proteins expressed by primary human lymphatic endothelial cells. [file 1476-4598-9-316-S8.PDF]

Table S1:

Primer Sequences (Forward/ Reverse) used for Quantitative PCR of major human HS core proteins.

HS Core Protein:	Forward Primer:	Reverse Primer:
Syndecan1	GGAGCAGGACTTCACCTTTG	TACAGCATGAAACCCACCAG
Syndecan2	GCTGCTCCAAAAGTGGAAAC	CAGCAATGACAGCTGCTAGG
Syndecan3	GAGCCTGACATCCCTGAGAG	CCCACAGCTACCACCTCATT
Syndecan4	GAGCCCTACCAGACGATGAG	CAGTGCTGGACATTGACACC
Glypican1	AGCGAGATGGAGGAGAACCT	CTGAGTACAGGTCCCGGAAG
Glypican2	TGACTACCTGCTCTGCCTCTC	GCTTCGCTGACCACATTTCT
Glypican3	GGCAAGTTATGTGCCCATTC	ATGTAGCCAGGCAAAGCACT
Glypican4	ATGGTGGCAGAGAGGCTAGA	GGAACGAGAAATTCGTCCAG
Glypican5	AAGCCCAGTCTGGAAATCCT	TCACAGTCCCCACTGACTTG
Glypican6	CACGTTTCAGGCCCTACAAT	GTTCCAGCATTCCTCCTCGT
Agrin	AACCTGGAGGAGGTGGAGTT	CTTCTTGCAGACGCAGGAC
Perlecan	CACTCGCTCCATCGAGTACA	GATGACCCTGAGCAGCATCT
Collagen-XVIII	CTGGGAGGCTCTGTTCTCAG	CACAGTAGCTCTCGGTCAGC
Beta-glycan	TGAAGTGACTGGACGAGACG	AGTGCGGAGATTCAGGACAT
CD44v3	CTGGGAGCCAAATGAAGAAA	AGCACTTCCGGATTTGAATG
Serglycin	GAGCACCCTGCTACATTTCC	CCGCGTAGGATAACCTTGAA
